# Kinesins in Cancer Drug Resistance: Mechanisms, Therapeutic Targeting, and Translational Potential

**DOI:** 10.3390/cancers18172872

**Published:** 2026-09-05

**Authors:** Yayun Tan, Ying Feng, Yueping Jiang, Meimei Li, Zhizhong Xie

**Affiliations:** 1 School of Pharmaceutical Science, University of South China, Hengyang 421001, China; tanyayun05@126.com; 2 Department of Clinical Pharmacy, Hunan University of Medicine General Hospital, Hunan University of Medicine, Huaihua 418000, China; 15897356771@163.com; 3 Department of Pharmacy, Xiangya Hospital, Central South University, Changsha 410008, China; jiangyueping@126.com

**Keywords:** kinesins, cancer drug resistance, mitotic kinesins, therapeutic targeting, biomarker potential, preclinical models

## Abstract

Drug resistance in cancer remains a major barrier to durable therapeutic benefits and limits the effectiveness of chemotherapy, targeted therapy, and combination treatment in multiple malignancies. Increasing evidence indicates that specific kinesin superfamily proteins contribute to tumor adaptation and therapeutic response in a context-dependent manner through their roles in mitotic regulation, intracellular transport, and stress-response pathways. Aberrant expression of multiple kinesin family members has been documented across diverse cancers and is frequently associated with aggressive clinicopathological features, poor prognosis, and resistance to treatment. However, expression alterations alone do not establish functional dependency, and mechanistic validation is required to distinguish true resistance drivers from adaptive tumor states. In this review, we summarize the classification, biological functions, and abnormal expression patterns of kinesins in cancer; discuss the major mechanisms through which they contribute to drug resistance; and examine strategies for targeting kinesins, including natural-product-derived direct inhibitors, small-molecule inhibitor development, rational combination approaches, and structure-guided and computational optimization strategies. We also evaluate the biomarker potential of kinesin dysregulation and the value of advanced preclinical models for mechanistic and translational investigations. Finally, we highlight the major challenges that hinder clinical translation, including target specificity, compensatory resistance, insufficient biomarker validation, and tumor heterogeneity. Future progress will require integration of functional genomics, multiomics profiling, and mechanism-guided therapeutic strategies to determine when kinesin inhibition represents a clinically actionable approach for resistant malignancies.

## Simple Summary

Cancer treatment often becomes less effective because tumor cells adapt and develop drug resistance. Kinesins are proteins that help cells divide and transport materials within the cell, but abnormal kinesin activity can also contribute to tumor progression and help some cancer cells adapt to treatment. This review examines how specific kinesins contribute to therapy adaptation and resistance in different cancer contexts. It also discusses whether kinesins can be used as markers to identify resistant tumors and whether blocking them could improve treatment. Current approaches include small-molecule inhibitors, compounds derived from natural products, rational drug combinations, and newer structure-based drug design methods. By bringing together evidence from different cancer types and experimental models, this review aims to clarify which kinesins have functional evidence supporting roles in treatment resistance, which represent potential biomarkers, and how these findings may guide the development of more selective therapies and patient stratification strategies.

## 1. Introduction

Drug resistance in cancer remains among the major obstacles to achieving durable therapeutic benefits in oncology. Although substantial progress has been made with respect to cytotoxic chemotherapy, molecularly targeted therapy, and immunotherapy, many tumors eventually develop intrinsic or acquired drug resistance through diverse mechanisms, including altered cell cycle control, enhanced DNA damage repair, adaptive stress responses, phenotypic plasticity, and compensatory signal rewiring. These resistance mechanisms are driven not only by canonical oncogenic pathways but also by intracellular transport systems and cytoskeletal regulators that coordinate mitosis, organelle dynamics, and spatiotemporal signal transduction. In this context, increasing evidence has revealed that specific kinesin superfamily proteins may contribute to malignant progression and therapy adaptation in a context-dependent manner. As microtubule-dependent motor proteins, kinesins regulate essential processes such as spindle assembly, chromosome segregation, vesicle trafficking, and intracellular cargo transport, thereby placing them at a strategic intersection among cell division, survival signaling, and therapeutic adaptation.

Among kinesin family members, mitotic kinesins such as *KIF11*, *KIF15*, *KIF18A*, *KIF23*, and *KIF2C* have received substantial attention because of their direct roles in spindle dynamics and chromosome fidelity, whereas cargo-transporting kinesins are being increasingly recognized for their involvement in receptor trafficking, stress adaptation, and signal maintenance. The aberrant expression of kinesins has been documented across multiple human malignancies and is frequently associated with aggressive clinicopathological features, poor prognosis, and resistance to chemotherapy, targeted therapy, and combined treatment modalities. However, elevated kinesin expression does not necessarily indicate functional dependency, and whether individual kinesins act as causal resistance drivers or represent adaptive consequences of tumor evolution requires mechanistic validation. Moreover, the therapeutic tractability of kinesins has stimulated the development of direct inhibitors, natural-product-derived modulators, structure-guided drug design strategies, and rational combination approaches. However, the current evidence remains fragmented across tumor types and mechanistic contexts, and the translational significance of kinesin dysregulation in drug resistance has not yet been synthesized in a sufficiently integrated manner.

In this review, we summarize the classification and functional characteristics of the kinesin superfamily, outline the expression patterns and clinical significance of kinesins in cancer, and discuss the major mechanisms through which specific kinesins contribute to therapeutic adaptation and resistance. Rather than simply cataloging kinesin alterations, we evaluate mechanistic evidence supporting kinesin dependency, distinguish functional resistance drivers from associated phenotypes, and discuss how these insights may inform therapeutic development. Furthermore, we examine current strategies targeting kinesins, including direct inhibitors, natural-product-derived compounds, and structure-guided optimization approaches, and evaluate the emerging biomarker potential and value of advanced preclinical models in this field. Finally, we discuss the major challenges that limit the clinical translation of these approaches and highlight future directions for developing kinesin-centered therapeutic and biomarker strategies for addressing resistant malignancies.

## 2. Overview of the Kinesin Family and Its Abnormal Expression in Cancer

### 2.1. Classification and Functional Overview of the Kinesin Family

The kinesin superfamily (KIF) comprises a diverse group of microtubule-dependent motor proteins that are essential for intracellular transport, mitotic progression, and the maintenance of cellular homeostasis. Structurally, kinesins share a conserved modular architecture consisting of a motor domain, a stalk or neck linker region, and a tail domain. The motor domain contains ATP-binding and microtubule-binding sites and serves as the mechanochemical core that converts the energy of ATP hydrolysis into directional movement along microtubule tracks. The stalk region generally mediates dimerization and structural flexibility, whereas the tail domain is highly variable and determines cargo specificity by binding diverse organelles, protein complexes, and RNA molecules [1,2]. This conserved yet adaptable architecture enables kinesins to couple a common motility machinery with highly specialized intracellular functions.

On the basis of the position of the motor domain and the movement direction along microtubules, the kinesin superfamily can be broadly classified into several major groups, including N-kinesins, C-kinesins, and M-kinesins, whereas the kinesin-13 family represents a functionally specialized microtubule-depolymerizing subgroup [3,4,5]. N-kinesins, in which the motor domain is located at the N-terminus, generally move toward the plus-end of microtubules and include many transport and mitotic kinesins [3,4]. C-kinesins possess a C-terminal motor domain and usually move toward the microtubule minus-end; this group includes kinesin-14 family members that participate in spindle assembly and microtubule organization [6]. M-kinesins, in which the motor domain is located in a central region, primarily regulate microtubule dynamics rather than long-range transport, particularly through microtubule depolymerization [5]. This classification helps explain the functional diversity of kinesins in transport, spindle regulation, and chromosome movement.

A major functional class of kinesins comprises mitotic kinesins, including *KIF11* (Eg5), *KIF15*, *KIF18A*, *KIF23*, and *KIF2C*/MCAK, which play essential roles in spindle assembly, centrosome separation, chromosome congression, chromosome segregation, and microtubule turnover. For example, *KIF11*, a kinesin-5 family member, is a homotetrameric plus-end-directed motor kinesin that is essential for establishing and maintaining spindle bipolarity through cross-linking and separating antiparallel microtubules [6]. *KIF15* functions in parallel with *KIF11* to support spindle assembly, whereas *KIF18A* regulates chromosome oscillation and positioning at the metaphase plate [7]. *KIF4A* is involved in chromosome condensation, chromosome-associated protein transport, and segregation during mitosis [8]. In addition, kinesin-8 family motor kinesins control microtubule length and kinetochore interactions, thereby ensuring appropriate chromosome alignment and segregation [9]. In contrast, kinesin-13 family members such as *KIF2C*/MCAK are specialized microtubule-depolymerizing motor kinesins that regulate spindle microtubule dynamics and chromosome attachment fidelity.

In addition to mitotic kinesins, cargo-transporting kinesins such as *KIF5B*, *KIF1A*, *KIF1B*, *KIF5A*, *KIF5C*, and *KIF13B* are essential for organelle positioning, vesicle trafficking, RNA transport, and signal transduction. *KIF5B*, a representative kinesin-1 family member, mediates the anterograde transport of mitochondria, vesicles, and other organelles [10]. *KIF1A* and *KIF13B*, which belong to the kinesin-3 family, support highly processive long-range cargo transport [11]. In addition, some kinesins participate in ciliary function, cytoskeletal organization, and signal regulation, further underscoring the broad functional diversity of this protein family [3].

In the context of cell cycle regulation, kinesins are indispensable for maintaining genomic stability. Appropriate spindle assembly, chromosome alignment, and chromosome segregation require precise cooperation between motor kinesins and nonmotor proteins. Alterations in kinesin expression or activity can disrupt these processes, resulting in spindle abnormalities, chromosome missegregation, aneuploidy, and chromosomal instability, all of which are hallmark features of cancer [3,6]. Accordingly, dysfunction or aberrant expression of these proteins has been associated with uncontrolled proliferation, enhanced cell migration, and facilitate tumor progression by destabilizing chromosome inheritance and altering intracellular transport programs.

Genomic analyses have revealed frequent copy number alterations and aberrant expression of kinesin genes across multiple solid tumors, and these alterations are significantly associated with poor overall survival. These findings highlight the clinical relevance of kinesin dysregulation; however, their biological consequences and therapeutic implications vary among individual kinesin members and tumor contexts. Because of their central roles in mitosis and intracellular logistics, kinesins have emerged as promising therapeutic targets. In particular, inhibitors directed against mitotic kinesins such as *Eg5*/*KIF11* aim to block spindle assembly or centrosome separation, thereby inducing mitotic arrest and apoptosis in cancer cells [3].

Taken together, the kinesin family can be classified according to the motor domain position, movement direction, and biological function, with distinct subfamilies specialized for cargo transport, spindle regulation, chromosome segregation, and microtubule depolymerization. Their structural diversity underlies their broad functional specialization, including intracellular transport, mitosis, and signal regulation. In cancer, kinesin dysregulation is frequently associated with chromosomal instability, altered proliferation, and changes in cellular behavior. However, the functional consequences of individual kinesin alterations depend on tumor lineage, cellular context, and experimental validation. Therefore, kinesins represent important regulators of cancer biology and potential therapeutic targets, but their specific roles require mechanistic investigation.

### 2.2. Cancer-Associated Kinesin Dysregulation and Clinical Associations

Bioinformatics analyses, multiomics profiling, and clinical sample validation have consistently shown that multiple kinesin family members are aberrantly expressed across various human malignancies. In particular, *KIF11*, *KIF18A*, *KIF2C*, *KIF4A*, *KIF18B*, and *KIFC1* are frequently overexpressed in breast cancer, lung cancer, colorectal cancer, liver cancer, ovarian cancer, glioblastoma, and other tumor types [12,13]. Integrated genomic, transcriptomic, and proteomic studies have further linked kinesin dysregulation to proliferative, cell-cycle, adhesion, and extracellular matrix-associated programs [1,14]. However, concordant alterations across multiple omics layers strengthen the evidence for cancer-associated dysregulation but do not, by themselves, establish functional dependence or a causal role in tumor progression. For example, integrated analyses have identified *KIF4A*, *KIF20A*, and *KIF11* as a three-gene prognostic signature in lung adenocarcinoma, supporting the value of kinesin-centered multiomics models for risk stratification [15,16,17]. In hepatocellular carcinoma, combined bulk RNA-seq and single-cell RNA-seq analyses have revealed that *KIF20A* is markedly overexpressed and associated with malignant progression and immune modulation, further extending the clinical relevance of kinesin dysregulation across tumor types [18]. These observations primarily establish expression and clinical associations rather than resistance-specific causality.

Clinically, elevated expression of kinesin family members is frequently associated with aggressive tumor phenotypes. High kinesin expression is reported to correlate with advanced tumor stage, a high histological grade, a high degree of lymph node metastasis, a high recurrence risk, and shorter overall survival (OS) or relapse-free survival (RFS), indicating their potential value for prognostic stratification. In several tumor types, multivariate analyses have further suggested that selected kinesins provide prognostic information beyond conventional clinicopathological variables [19,20]. This pattern is also illustrated by *KIF26B* in colorectal cancer, as elevated *KIF26B* expression is associated with tumor size, AJCC stage, T stage, N stage, and differentiation status and independently predicts poor overall survival [21]. Similar clinicopathological associations have been reported for *KIF20B* in breast cancer, as high *KIF20B* expression is correlated with advanced nodal stage and independently predicts unfavorable prognosis [22]. A broader summary of cancer-associated kinesin expression patterns and clinicopathological or prognostic associations is provided in Supplementary Table S1.

The direction and clinical consequences of kinesin dysregulation are nevertheless context dependent. *KIFC1*, a kinesin-14 family member, is frequently overexpressed across multiple tumor types and is consistently associated with adverse clinical outcomes in large-scale datasets such as The Cancer Genome Atlas (TCGA) [13]. For example, increased *KIFC1* expression has been associated with aggressive clinicopathological features and unfavorable outcome across several malignancies [13,23,24,25,26,27,28]. In contrast, not all kinesins are overexpressed in cancer. *KIF17* is significantly downregulated in breast cancer, and its reduced expression is associated with poor clinical outcomes, suggesting that *KIF17* may have a tumor-suppressive role in this context [29]. A similar context-dependent pattern has been reported for *KIF3A* in NSCLC, where reduced *KIF3A* expression was associated with poor survival and enhanced proliferative and metastatic behavior, illustrating that the biological consequences of kinesin dysregulation cannot be generalized across family members or tumor lineages [30].

Importantly, recurrent expression or prognostic associations should not be interpreted as evidence that a kinesin is a common molecular driver across tumor types. Such associations may reflect proliferative activity, tumor grade, lineage-specific biology, or other correlated malignant programs. Functional relevance therefore needs to be established separately in each tumor and treatment context.

Taken together, cancer-associated kinesin dysregulation is recurrent across diverse malignancies and is frequently associated with tumor stage, metastatic behavior, recurrence, and survival. However, these expression and prognostic observations constitute a distinct evidence domain from therapeutic-resistance causality. Increased kinesin expression in a resistant tumor, residual disease, or a poor-outcome cohort may reflect proliferation, tumor aggressiveness, or treatment-associated cell-state changes without being functionally required for resistance. Establishing a kinesin as a resistance driver therefore requires evidence that genetic or pharmacological perturbation alters drug response, ideally in an established resistant model, with further support from mechanistic rescue or epistasis, in vivo resistance reversal, or treatment-response clinical data. Accordingly, expression and clinicopathological associations are summarized separately in Supplementary Table S1, whereas resistance-specific functional evidence is critically evaluated in Table 1. This distinction provides the basis for the mechanistic discussion of kinesin-mediated therapeutic resistance in the following section.

## 3. Mechanisms of Kinesin-Associated Therapeutic Resistance in Cancer

**Figure 1:**
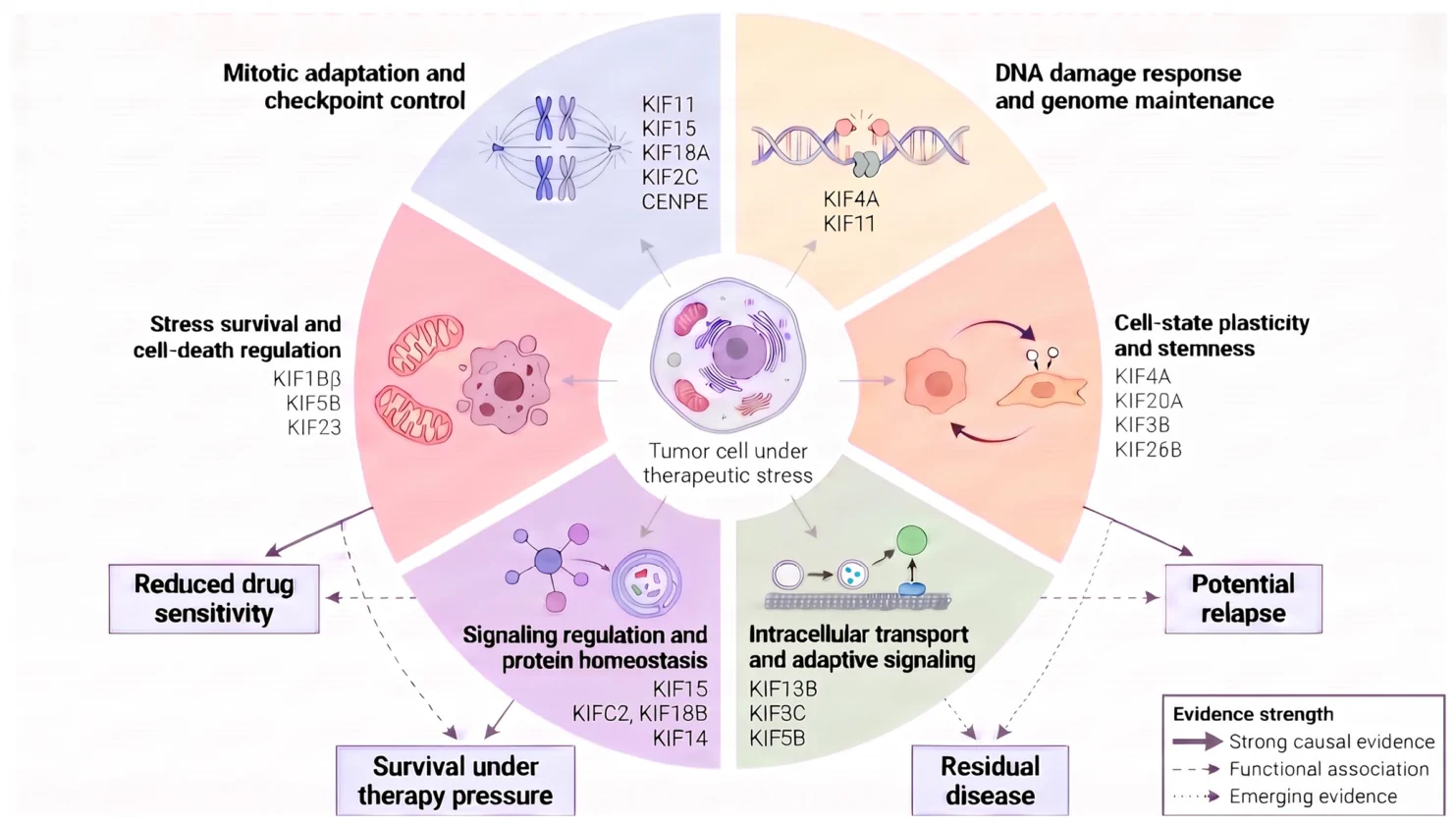
Context-dependent mechanisms of kinesin-associated therapeutic adaptation and drug resistance. Kinesin dysregulation is associated with therapeutic adaptation through multiple biological processes, including mitotic regulation, DNA damage response, cell-state plasticity, stress survival, intracellular transport, and signaling regulation. Different colors represent distinct functional categories of kinesin-mediated resistance mechanisms. Arrows indicate different levels of evidence, including strong causal evidence, functional association, and emerging evidence. Evidence strength is classified according to experimental validation, distinguishing functionally validated mechanisms from association-level observations and emerging hypotheses. Created in BioRender. Li, M. (2026) https://BioRender.com/nyz0r72 (accessed on 22 August 2026).

Kinesin family members contribute to therapeutic resistance through multiple interconnected mechanisms, including the regulation of mitotic fidelity, DNA damage handling, stress-adaptive cell death programs, and stemness-associated phenotypes, as well as the intracellular trafficking of signaling components. As microtubule-based motor proteins, kinesins are strategically positioned to coordinate vesicular trafficking, organelle dynamics, chromosome segregation, and signal transduction, thereby enabling tumor cells to survive therapeutic stress. Increasing evidence indicates that kinesin-therapeutic resistance extends beyond classic antimitotic adaptation and involves genome maintenance, cancer stemness, metabolic and redox adaptation, regulated cell-death programs, and adaptive signal rewiring, all of which can promote tumor persistence and relapse. Emerging studies further implicate transcriptional and epigenetic regulation, mitochondrial homeostasis, ferroptosis, and tumor-microenvironmental remodeling in kinesin-associated treatment responses. The major mechanisms through which kinesins contribute to therapeutic resistance are summarized in Figure 1.

Importantly, increased kinesin expression in resistant tumors does not by itself establish a causal resistance function. Evidence is stronger when genetic or pharmacological perturbation alters drug response, particularly in an established resistant model, and is further strengthened by gain-of-function sufficiency, mechanistic rescue or epistasis, in vivo resistance reversal, or treatment-response clinical evidence. Accordingly, the following sections distinguish established resistance dependencies from drug-sensitivity modifiers and association-level observations (Table 1).

### 3.1. Regulation of Cell Cycle Checkpoints, Microtubule Regulation, and Mitotic Fidelity

A major mechanism of kinesin-associated therapeutic response involves the regulation of mitotic spindle assemblies, chromosome congression, and cell cycle checkpoints. *KIF11* (Eg5), a plus-end-directed kinesin-5 motor, is essential for centrosome separation and bipolar spindle formation [15]. Pharmacological inhibition of *KIF11* induces monopolar spindle formation, activates the spindle assembly checkpoint (SAC), and leads to mitotic arrest and cell death [17]. Thus, *KIF11* is an attractive antimitotic target. However, cancer cells can develop resistance by activating compensatory pathways, most notably through upregulation of the expression of *KIF15*, a kinesin-12 family member that cooperates with dynein to maintain spindle bipolarity independent of *KIF11* [54]. This functional redundancy enables tumor cells to continue mitosis even in the presence of *KIF11* inhibitors. This phenomenon is particularly relevant to resistance against kinesin-directed antimitotic therapy and illustrates how compensation between mitotic motors can limit single-target inhibition.

Mitotic kinesins have also been reported to be involved in resistance to microtubule-targeting agents. In triple-negative breast cancer, *KIF2C* promotes paclitaxel resistance and broader cross-resistance to multiple microtubule-targeting agents by preferentially depolymerizing polyglutamylated tubulin even in the presence of paclitaxel, while pharmacologic inhibition of *KIF2C* restores drug sensitivity [32]. Genetic *KIF2C* depletion and pharmacological inhibition with 7S9 resensitized resistant models, including chemoresistant tumors in vivo, providing strong causal evidence for *KIF2C*-dependent resistance. Similarly, ectopic overexpression of MCAK confers resistance to paclitaxel and epothilone A, suggesting that enhanced microtubule depolymerase activity can counteract the effects of microtubule-stabilizing drugs [33]. Suppression of ectopic MCAK expression reverses this induced resistant phenotype, providing complementary gain-of-function evidence. In prostate cancer, *KIF14* was identified as a driver of cross-resistance between docetaxel and cabazitaxel, and its silencing restored taxane sensitivity and reduced *Akt* phosphorylation [34]. In ovarian cancer, *KIF26B* knockdown reduced paclitaxel resistance, altered microtubule polymerization dynamics, and promoted apoptosis in resistant cells, further linking kinesin dysregulation to anti-microtubule drug tolerance [35]. Because both studies directly perturbed kinesins in experimentally established resistant cells, they provide stronger resistance-specific evidence than expression-based studies alone.

Importantly, mitotic dysregulation has a dual effect on chemoresistance. On the one hand, preserved mitotic fidelity enables survival under antimitotic stress conditions; on the other hand, chronic spindle defects and chromosome missegregation can generate aneuploid, genetically diverse subclones with increased adaptive potential. However, this latter relationship should be interpreted cautiously because chromosomal instability and treatment resistance can coexist without establishing a direct causal link. Thus, kinesin-mediated control of mitosis not only affects acute drug responses but also may contribute to long-term clonal evolution during treatment.

### 3.2. Apoptosis, Protective Autophagy, and Stress-Survival Programs

Kinesins also influence the balance between apoptosis and autophagy, two major determinants of tumor cell fate under chemotherapeutic stress conditions. Although *KIF1B*β- and *KIF5B*-related transport systems participate in mitochondrial and organelle-associated cell-death processes [55,56], these studies primarily establish fundamental stress-response biology rather than direct chemotherapy-resistance mechanisms. Their mitochondrial functions are therefore discussed in greater detail in Section 3.8.

Conversely, more direct kinesin-specific resistance evidence has been obtained in chronic myeloid leukemia, where *KIF23* knockdown suppressed autophagy and restored imatinib sensitivity through the inactivation of Wnt/β-catenin signaling [36]. *KIF23* is elevated in imatinib-resistant K562/R cells, and its depletion decreases the imatinib IC50 and increases apoptosis. Importantly, reactivation of autophagy with rapamycin or restoration of Wnt/β-catenin signaling with BML-284 partially rescues the effects of *KIF23* depletion, providing pathway-level evidence that *KIF23* supports resistance through Wnt/β-catenin-dependent protective autophagy [36].

Taken together, the current evidence suggests that kinesins may contribute to chemoresistance not only through mitotic or trafficking functions but also by influencing stress-adaptive cell death programs. However, compared with the evidence for mitotic regulation, the direct mechanistic basis linking individual kinesins to coordinated apoptosis–autophagy rewiring remains less well defined. At present, *KIF23* represents one of the better-supported resistance-specific examples in this category.

### 3.3. DNA Damage Response and Genome Maintenance

Enhanced DNA damage repair (DDR) is another important mechanism through which cancer cells resist chemotherapy, particularly genotoxic agents such as platinum compounds. Kinesins may contribute to this process by regulating chromatin dynamics, repair factor localization, and the microtubule-dependent organization of DNA repair events. Direct evidence has been reported for the role of *KIF4A* in non-small cell lung cancer, where cisplatin treatment increases *KIF4A* expression, and depletion of *KIF4A* enhances cisplatin-induced cell cycle arrest and cytotoxicity while suppressing *BRCA2* and *Rad51* focus formation and limiting *PARP1* activation [37]. However, because this study did not employ a pre-established cisplatin-resistant derivative, it demonstrates *KIF4A*-dependent cisplatin sensitization rather than reversal of acquired resistance. Similarly, in colorectal cancer, *KIF11* knockdown increases oxaliplatin sensitivity by enhancing DNA damage and apoptosis, suggesting that kinesin dysregulation may influence cellular responses to DNA-damaging therapy [38]. Mechanistic rescue supports involvement of p53/GSK3β-associated signaling, but an established oxaliplatin-resistant model was not used. *KIF11* should therefore be considered a functional modifier of platinum sensitivity in this setting rather than a fully established acquired-resistance driver.

Taken together, the current evidence suggests that selected kinesins can modulate sensitivity to platinum compounds not only by affecting mitotic function but also by influencing DNA damage handling and genome maintenance under therapeutic stress conditions. Resistance causality, however, should be distinguished from sensitization unless kinesin perturbation directly reverses a pre-existing resistant phenotype.

### 3.4. Cancer Stemness, Metabolic Reprogramming, and Phenotypic Plasticity

Kinesins may also contribute to chemotherapy resistance by sustaining cancer stem cell (CSC) properties and metabolic or phenotypic plasticity, both of which are strongly associated with relapse, metastasis, and drug tolerance. Metabolic reprogramming can enable tumor cells to redistribute energetic and biosynthetic resources under therapeutic stress and thereby contribute to adaptive survival [57]. CSCs are characterized by self-renewal, quiescence, and intrinsic resistance to conventional therapies. Recent studies have provided functional support for this concept. For example, in glioma, *KIF11* promotes stemness, cell proliferation, and chemoresistance, indicating that kinesin dysregulation may support stem-like tumor states during treatment [39]. *KIF11* gain- and loss-of-function alters 5-FU response together with stemness markers including *KLF4*, *NANOG*, *OCT4*, *LIN28*, and CD133. However, because a pre-established 5-FU-resistant model and resistance-specific rescue experiments were not used, the study supports drug-sensitivity modulation rather than acquired-resistance reversal. In oxaliplatin-resistant colon cancer, *KIF4A* increases glycolysis, cancer stemness, and drug resistance, and these effects can be reversed by glycolysis inhibition [40]. *KIF4A* knockdown or overexpression alters oxaliplatin response, glycolysis inhibition attenuates the *KIF4A*-dependent phenotype, and resistant xenograft experiments provide in vivo support. This represents substantially stronger evidence for a metabolic kinesin-dependent resistance mechanism. In addition, *KIF20A* has been identified as a glioma biomarker associated with the TMZ response and was shown to influence malignant behavior partly through EMT-related regulation [41]. However, *KIF20A* itself was not directly perturbed during TMZ treatment; this study therefore provides association-level rather than causal resistance evidence.

Together, these findings suggest that kinesins may contribute to resistance by sustaining stemness-associated metabolic programs and EMT-linked plasticity, although direct mechanistic evidence is available only for a limited number of tumor contexts. Additional links between stemness and ferroptosis resistance are discussed in Section 3.8.

### 3.5. Cargo Trafficking, Receptor Recycling, and Adaptive Signaling

Adaptive signaling can depend on the intracellular localization, recycling, and persistence of membrane receptors and signaling complexes. Because kinesins mediate microtubule-dependent cargo transport, altered motor activity may sustain signaling during therapeutic stress without directly altering the abundance of the corresponding oncogene. Kinesin motor proteins are well positioned to influence this process because they mediate the anterograde transport of various components along microtubules. A closely related mechanism was reported in lung squamous cell carcinoma, where VASH2-driven tubulin detyrosination enhanced the binding of *KIF3C* to microtubules and promoted *KIF3C*-dependent endosomal recycling of EGFR, thereby prolonging PI3K/*Akt*/mTOR signaling and contributing to paclitaxel resistance [42]. *KIF3C* depletion attenuated VASH2-induced resistance to paclitaxel, cisplatin, and gemcitabine and reduced receptor-recycling-associated signaling. However, direct *KIF3C*-dependent resensitization was not demonstrated in the independently established SK-MES-1 paclitaxel-resistant derivative, and the in vivo chemotherapy experiments principally validated the upstream VASH2 program. Thus, this study provides functional but incomplete evidence for a *KIF3C*-specific acquired-resistance mechanism. Overall, current findings support the concept that kinesin-dependent cargo transport may facilitate receptor recycling and adaptive signaling under therapeutic conditions, although the extent of this mechanism likely depends on the tumor context and the specific kinesin involved.

### 3.6. Oncogenic Signaling and Protein Stability

In addition to receptor trafficking, kinesins may contribute to drug resistance by helping sustain oncogenic signaling outputs and the stability of resistance-associated proteins. Consistent with this concept, *KIF14* colocalizes with *AKT* at the plasma membrane in triple-negative breast cancer; furthermore, *KIF14* depletion increases docetaxel sensitivity while reducing *AKT* phosphorylation, suggesting that kinesin-dependent spatial regulation of signaling effectors may contribute to chemotherapy resistance [43]. *KIF14* re-expression and constitutively active *AKT* rescue the sensitized phenotype, strengthening the mechanistic connection; however, the study did not use an established acquired docetaxel-resistant derivative. This mechanism has been directly demonstrated in several therapy-resistant tumor settings. In castration-resistant prostate cancer, *KIF15* directly binds AR/AR-V7 and prevents its degradation by enhancing its association with U*SP1*4, thereby sustaining AR signaling and increasing enzalutamide resistance [44]. Reciprocal AR-dependent transcriptional induction of *KIF15* establishes a positive-feedback loop, and genetic or pharmacological *KIF15* inhibition suppresses enzalutamide-resistant models in vivo. Similarly, in hormone receptor-positive/HER2-negative breast cancer, *KIFC2* stabilizes *CDK4* by enhancing its interaction with *USP9X*, and depletion of *KIFC2* increases sensitivity to tamoxifen and *CDK4*/6 inhibitors [45]. *CDK4* re-expression partially rescues resistance, and the mechanism is supported by resistant cell lines, xenografts, and patient-derived organoids, providing strong evidence for a *KIFC2*-dependent protein-stability resistance pathway. The *KIF18B*–*SP1*/*DNMT3B*–*PARPBP* axis [46] and the *NSUN2*/*YBX1*–*SERPINB5* motor-associated program [48] involve distinct epigenetic or RNA-regulatory mechanisms and are therefore discussed in Section 3.7. The gastric taxane-associated multi-KIF signature is retained as association-level evidence in Table 1 rather than as a defined signaling mechanism. Additionally, studies have suggested that kinesins can reinforce resistance-associated signaling networks. In head and neck squamous cell carcinoma, *SHCBP1* interacts with *KIF23* and cooperatively promotes cisplatin resistance through the activation of PI3K/*Akt*, ERK1/2, NF-κB-p65, and Wnt/β-catenin signaling [49]. *KIF23* depletion attenuates *SHCBP1*-induced cisplatin resistance, although direct *KIF23*-mediated resensitization of the established cisplatin-resistant derivatives was not demonstrated. In hepatocellular carcinoma, *KIF14* was identified as a mediator of acquired sorafenib resistance through an *AKT*-*ETS1*-*KIF14* positive feedback loop, and *KIF14* silencing restored sorafenib sensitivity in resistant tumors [50]. Chronic sorafenib exposure induces this signaling circuit, and *KIF14* depletion restores drug response in established resistant cells and xenografts, supporting a genuine acquired-resistance dependency. In gemcitabine-resistant cholangiocarcinoma, suppression of *KIF18A* reduced colony formation, migration, and survival while downregulating PI3K/*Akt*/mTOR, NF-κB, and Bcl-2 signaling [51]. Importantly, however, *KIF18A* depletion did not significantly restore gemcitabine sensitivity. Thus, *KIF18A* appears to support the fitness of gemcitabine-resistant cells without being demonstrated as a direct mediator of the drug-resistant phenotype.

Taken together, these findings suggest that selected kinesins may help sustain oncogenic signaling outputs and resistance-associated survival programs in a context-dependent manner. However, compared with the evidence for mitotic regulation, the direct mechanistic basis linking individual kinesins to the maintenance of the stability of broad oncogenic proteins remains less well defined. The strongest evidence is currently provided by defined protein-stability or feedback circuits in which kinesin perturbation directly resensitizes resistant models, whereas signaling associations without resistance reversal should be interpreted more cautiously.

### 3.7. Epigenetic, Transcriptional, and RNA-Based Regulation of Kinesin-Associated Therapeutic Resistance

Kinesin dysregulation during tumor progression and therapeutic adaptation can arise through transcriptional, chromatin, DNA-methylation, RNA-modification, and non-coding RNA mechanisms. However, upstream regulation of a kinesin should not automatically be equated with a kinesin-dependent resistance mechanism; the critical question is whether perturbation of the regulated kinesin alters the treatment-resistant phenotype.

#### 3.7.1. Transcriptional and Chromatin-Mediated Regulation

In cervical cancer, *JNK1*/*c-Jun* signaling directly activates *KIF18A* transcription through *c-Jun* binding to the *KIF18A* promoter [58]. Although this establishes a transcriptional mechanism controlling *KIF18A*, treatment resistance was not directly evaluated. Similarly, *ANCCA*/ATAD2 coordinates estrogen-dependent transcription of *KIF4A*, *KIF15*, *KIF20A*, and *KIF23* in ER-positive breast cancer [59]. This coordinated kinesin program is associated with adverse relapse-related outcomes, but individual kinesin dependencies during endocrine treatment were not demonstrated.

Chromatin remodeling provides another regulatory layer. In metastatic castration-resistant prostate cancer, *NSD2*-dependent increases in H3K36me2, loss of H3K27me3, and inactive-to-active chromatin compartment switching are associated with activation of *KIF18A* and other downstream genes [60]. Functional analyses support a contribution of *KIF18A* to CRPC growth, but direct reversal of androgen-deprivation or AR-targeted resistance by *KIF18A* inhibition was not established.

A more resistance-specific transcriptional circuit has been identified in gastric cancer. *YY1* transcriptionally activates *KIF15*, whereas *KIF15* interacts with and stabilizes the antioxidant protein *PRDX1*, thereby maintaining mitochondrial redox homeostasis and promoting cisplatin resistance [61]. Genetic perturbation, rescue experiments, and in vivo chemoresistance studies support the functional relevance of this *YY1*–*KIF15*–*PRDX1* circuit.

#### 3.7.2. DNA Methylation-Dependent Regulation

In oxaliplatin-resistant colorectal cancer, *KIF18B* interacts with *SP1* and reduces *DNMT3B* recruitment to the *PARPBP* promoter, resulting in promoter hypomethylation and increased *PARPBP* expression [46]. Manipulation of the *KIF18B*–*PARPBP* axis alters the resistant phenotype, supporting a functional connection between a kinesin and an epigenetically maintained oxaliplatin-resistance program.

#### 3.7.3. RNA Modification and Non-Coding RNA Regulation

RNA modifications can also establish kinesin-associated treatment-resistant states. In hepatocellular carcinoma, *METTL3*-mediated m6A modification stabilizes the long non-coding RNA *KIF9-AS1*, which promotes U*SP1*-dependent *SHOX2* stabilization, stemness, and sorafenib resistance [62]. Because *KIF9-AS1* is a non-coding transcript rather than a kinesin motor protein, this represents a kinesin-associated epitranscriptomic mechanism rather than direct regulation of KIF9 protein.

Non-coding RNAs can also directly control kinesin proteins. In oxaliplatin-resistant colorectal cancer, lncRNA *AC105118.1* relieves *miR-378a-3p*-mediated repression of *KIF26B*, increasing glycolytic activity and resistance [63]. *KIF26B* restoration functionally rescues the effects of the miRNA axis, and the upstream pathway is supported in vivo. Similarly, *miR-127-3p* suppresses *KIF3B* in docetaxel-resistant triple-negative breast cancer, whereas *KIF3B* restoration partially rescues stemness and docetaxel resistance [64].

In cervical cancer, *NSUN2*/*YBX1*-mediated m5C stabilization of *SERPINB5* promotes paclitaxel and vincristine resistance and is accompanied by increased expression of *CENPE*, *KIF16B*, and *KIF21A* [48]. However, these individual motors were not functionally perturbed during treatment; therefore, this represents an indirect kinesin-associated resistance program rather than evidence that these kinesins independently mediate resistance.

Hypoxia-induced *ALYREF*-mediated m5C regulation further activates a *KIF20A*/*BUB1* program that increases ferroptosis resistance in cervical cancer [65]. The upstream RNA-regulatory mechanism is considered here, whereas its implications for ferroptotic vulnerability are discussed in Section 3.8.

Collectively, epigenetic and RNA-based regulation can generate kinesin-associated resistant states, but the evidence ranges from regulation of kinesin expression to direct resistance-rescue mechanisms. These distinctions are summarized in Table 2.

### 3.8. Mitochondrial Trafficking, Redox Homeostasis, and Alternative Cell Death Vulnerabilities

Mitochondrial and redox adaptation represent increasingly recognized components of tumor-cell survival under therapeutic stress. Recent evidence further demonstrates that structural remodeling of mitochondria and associated metabolic reprogramming can directly support targeted-therapy resistance, as exemplified by Mic60-dependent mitochondrial remodeling in EGFR-TKI-resistant lung cancer [66]. In addition to their mitotic and cargo-transport functions, kinesin-associated transport systems regulate organelle positioning, mitochondrial quality control, oxidative homeostasis, and stress-associated cell-death pathways. Importantly, the available evidence ranges from fundamental mitochondrial biology to direct resistance-specific validation.

#### 3.8.1. Kinesin-Dependent Mitochondrial Trafficking and Organelle Stress Adaptation

*KIF1B*- and *KIF5B*-related transport systems have been implicated in mitochondrial and lysosomal positioning, mitophagy-associated processes, and stress-induced cell death [55,56,67]. *KIF1B*β, for example, regulates calcineurin-dependent mitochondrial fission and apoptosis and cooperates with YME1L1 in mitochondrial apoptotic signaling [68,69]. These studies provide mechanistic background but do not directly establish drug resistance.

More direct treatment-specific evidence is provided by kinesin light chain 4 (KLC4). In radioresistant R-H460 lung cancer cells, KLC4 depletion enhances radiosensitivity and apoptosis and induces mitochondrial dysfunction with altered mitochondrial calcium handling [70]. KLC4 depletion also enhances the response to radiation in xenografts, providing direct evidence that the kinesin transport machinery can contribute to mitochondrial radioresistance.

#### 3.8.2. Metabolic and Redox Adaptation

As discussed in Section 3.4, *KIF4A*-dependent glycolytic reprogramming sustains oxaliplatin resistance [40], illustrating how metabolic adaptation can cooperate with kinesin-dependent treatment survival.

A more direct redox mechanism has been identified in gastric cancer. *KIF15* interacts with and stabilizes *PRDX1*, lowers oxidative stress, and preserves mitochondrial function [61]. Rescue experiments and in vivo chemoresistance models support its role in cisplatin resistance. Additional studies linking *KIF15* to ROS-dependent cancer stemness in hepatocellular carcinoma and suppression of ROS-mediated apoptosis in gastric cancer further support a broader redox-regulatory role, although their treatment-resistance evidence is less direct.

#### 3.8.3. Kinesins and Ferroptosis-Associated Therapeutic Vulnerabilities

Ferroptosis represents one of the clearest emerging non-mitotic pathways connecting kinesins with therapeutic response. In oxaliplatin-resistant colorectal cancer, *KIF20A* suppresses ferroptosis through the *NUAK1*–Nrf2–*GPX4* pathway, whereas *KIF20A* inhibition restores oxaliplatin sensitivity and promotes ferroptotic cell death [71]. Mechanistic rescue and resistant xenograft experiments provide strong causal support.

Similarly, *KIF4A* is increased in temozolomide-resistant glioblastoma and suppresses ferroptosis through the *CHMP4B*/*GPX4* axis [72]. *KIF4A* depletion increases ferroptotic vulnerability and TMZ sensitivity, mechanistic rescue implicates *CHMP4B*, and xenograft studies support enhanced TMZ response following *KIF4A* inhibition.

In oral squamous cell carcinoma, *KIF20A* inhibits *TRIM21*-dependent ubiquitination of *DHX9*, thereby stabilizing *DHX9* and increasing *SOX2* expression, jointly enhancing cancer stemness and ferroptosis resistance [73]. Pharmacological inhibition increases cisplatin sensitivity, although the multi-target properties of the inhibitor preclude assigning this effect exclusively to *KIF20A*.

Hypoxia-induced *ALYREF*-mediated m5C regulation of the *KIF20A*/*BUB1* axis provides an additional link between RNA regulation and ferroptosis resistance in cervical cancer [65]. Additional studies implicate *KIF14*–*AKT* signaling in ferroptosis sensitivity in triple-negative breast cancer and *KIF20A*–*HMOX1*/*SLC7A11*/*GPX4* signaling in hepatocellular carcinoma, although direct drug-resistance evidence differs among these models.

#### 3.8.4. Evidence Boundaries and Therapeutic Implications of Mitochondrial and Redox Mechanisms

The evidence linking kinesins to mitochondrial and redox adaptation is therefore heterogeneous. *KIF1B*β/*KIF5B* studies largely provide biological plausibility, KLC4 directly links mitochondrial dysfunction to radioresistance, the *YY1*–*KIF15*–*PRDX1* circuit connects antioxidant maintenance with cisplatin resistance, and selected *KIF20A* and *KIF4A* studies establish ferroptosis-dependent chemotherapy resistance. These pathways suggest opportunities for combining kinesin-directed strategies with oxidative-stress or ferroptosis-inducing therapies, but the physiological importance of mitochondrial transport and redox homeostasis necessitates careful evaluation of tumor selectivity and normal-tissue toxicity.

### 3.9. Tumor-Microenvironment, Immune, and Vascular Functions

Most kinesin-resistance studies have focused on tumor-cell-intrinsic mechanisms. However, therapeutic response is also shaped by interactions among malignant cells, immune populations, endothelial cells, and fibroblasts. Current evidence supporting kinesin-dependent TME resistance is considerably less mature than tumor-cell-intrinsic evidence, and immune or stromal associations should therefore be distinguished from cell-type-specific functional resistance mechanisms.

#### 3.9.1. Immune Modulation and Tumor–Immune Interactions

*KIFC1* provides a clinically relevant example in bladder cancer. *KIFC1*-positive tumors are associated with PD-L1 positivity, and high *KIFC1* expression in the IMvigor210 cohort was associated with favorable outcome following atezolizumab treatment [26]. However, *KIFC1* perturbation was not shown to regulate PD-L1 or checkpoint-blockade response. Thus, these findings represent clinical immunotherapy-response association rather than a causal *KIFC1*-dependent immune-resistance mechanism.

More direct evidence of tumor–immune remodeling has recently been reported for *KIF20A* in gastric adenocarcinoma, where *KIF20A* promotes tumor-cell proliferation and migration while facilitating macrophage M2 polarization and immune evasion [74]. Whether this macrophage-remodeling phenotype directly alters immune-checkpoint blockade response remains unknown.

*KIF20A* can also be therapeutically exploited as an immunogenic tumor-associated antigen. In hepatocellular carcinoma, a *KIF20A*-based thermosensitive hydrogel vaccine enhances PD-L1 blockade, increases dendritic-cell maturation, and promotes CD8^+^ T-cell infiltration in CDX and immune-humanized PDX models [75]. Importantly, this demonstrates therapeutic exploitation of *KIF20A* as an antigen rather than endogenous *KIF20A*-mediated checkpoint resistance.

#### 3.9.2. Endothelial and Vascular Kinesin Functions

Endothelial-specific Kif3a deletion provides one of the strongest cell-type-specific TME examples. In ovarian cancer models, loss of endothelial Kif3a disrupts primary cilia, promotes endothelial-to-mesenchymal transition, activates *EphA2*-associated signaling, and induces vascular abnormalities and tumor-supportive microenvironmental remodeling [76]. However, therapy-response experiments were not performed. *KIF3A* should therefore be regarded as a functionally validated regulator of the vascular TME rather than an established vascular resistance driver.

Single-cell analyses in breast cancer additionally identify *KIF20A* enrichment in endothelial populations [77]. Because this observation derives from transcriptional localization rather than endothelial-specific perturbation, it represents association-level evidence.

#### 3.9.3. Stromal Remodeling and Resistance-Associated Programs

A recent breast cancer study links *KIF20A* with both treatment outcome and stromal cell states. High *KIF20A* expression was associated with increased recurrence after radiotherapy and chemotherapy and with poorer disease-free survival. In patients receiving anthracycline- and/or taxane-containing neoadjuvant chemotherapy, the pathological complete response rate was lower in *KIF20A*-high tumors than in *KIF20A*-low tumors (20% versus 37%) [77].

Single-cell analysis from the same study identified *KIF20A* enrichment in fibroblast and endothelial populations, where *KIF20A* was co-expressed with the multidrug-resistance-associated transporters *ABCB1*, *ABCC1*, and *ABCG2*. However, *KIF20A* was not selectively manipulated in stromal cells, and no experiment demonstrated that *KIF20A* regulates these transporters or that stromal *KIF20A* depletion restores drug sensitivity. These observations therefore identify a resistance-associated stromal *KIF20A* state rather than a causal stromal resistance mechanism.

Direct evidence for kinesin-dependent regulation of cancer-associated fibroblast-mediated resistance remains sparse. Future studies should combine fibroblast- or endothelial-specific perturbation with coculture systems, spatial profiling, organotypic models, and in vivo treatment-response experiments to determine whether stromal kinesin dysregulation represents an actionable dependency or a secondary marker of treatment-induced remodeling.

#### 3.9.4. Evidence Boundaries and Therapeutic Implications

Collectively, current TME-related evidence encompasses clinical treatment-response association (*KIFC1*), tumor–immune remodeling (*KIF20A*), antigen-directed immunotherapy (*KIF20A*), cell-type-specific vascular remodeling (*KIF3A*), and resistance-associated fibroblast/endothelial states (*KIF20A*). These observations are biologically relevant but remain qualitatively different from established resistance models in which kinesin perturbation directly restores therapeutic sensitivity.

Accordingly, TME-associated kinesin biology should currently be regarded as an emerging dimension of therapeutic resistance rather than a fully established resistance mechanism. Lineage-specific genetic perturbation in immune-competent treatment models will be required to determine whether immune-, endothelial-, or fibroblast-specific kinesin dependencies can be therapeutically exploited.

Taken together, kinesin-associated therapeutic resistance is mechanistically heterogeneous and highly context dependent. The strongest causal evidence derives from studies in which kinesin perturbation reverses resistance in established models and is supported by mechanistic rescue and in vivo validation, including selected *KIF2C*-, *KIF4A*-, *KIF14*-, *KIF15*-, *KIF20A*-, and *KIFC2*-dependent pathways. By contrast, epigenetic, mitochondrial, ferroptotic, and TME-associated mechanisms span a continuum from functionally validated resistance circuits to emerging associations that remain incompletely tested. This distinction is therapeutically important: recurrent kinesin overexpression does not necessarily indicate a targetable resistance dependency. Rather, kinesin-directed intervention is most likely to succeed when a treatment-specific functional dependence can be experimentally demonstrated and subsequently identified through predictive biomarkers.

## 4. Strategies Targeting Kinesins to Overcome Drug Resistance: Direct Inhibition, Selective Vulnerabilities, and Combination Approaches

Kinesins have emerged as potential therapeutic targets for overcoming drug resistance in cancer because of their central roles in mitotic progression, intracellular transport, and stress adaptation. Among them, mitotic kinesins, particularly *KIF11*/Eg5, whose inhibition can disrupt bipolar spindle assembly and induce mitotic catastrophe, have attracted substantial attention. Additionally, other kinesin family members, such as *KIF18A* and *KIFC1*/HSET, and rational combination strategies designed to increase efficacy and delay resistance have been investigated. However, the development of kinesin-targeted therapies is complicated by acquired resistance, functional compensation, and the challenge of selectively targeting pathogenic kinesin functions without disrupting essential physiological processes.

**Figure 2:**
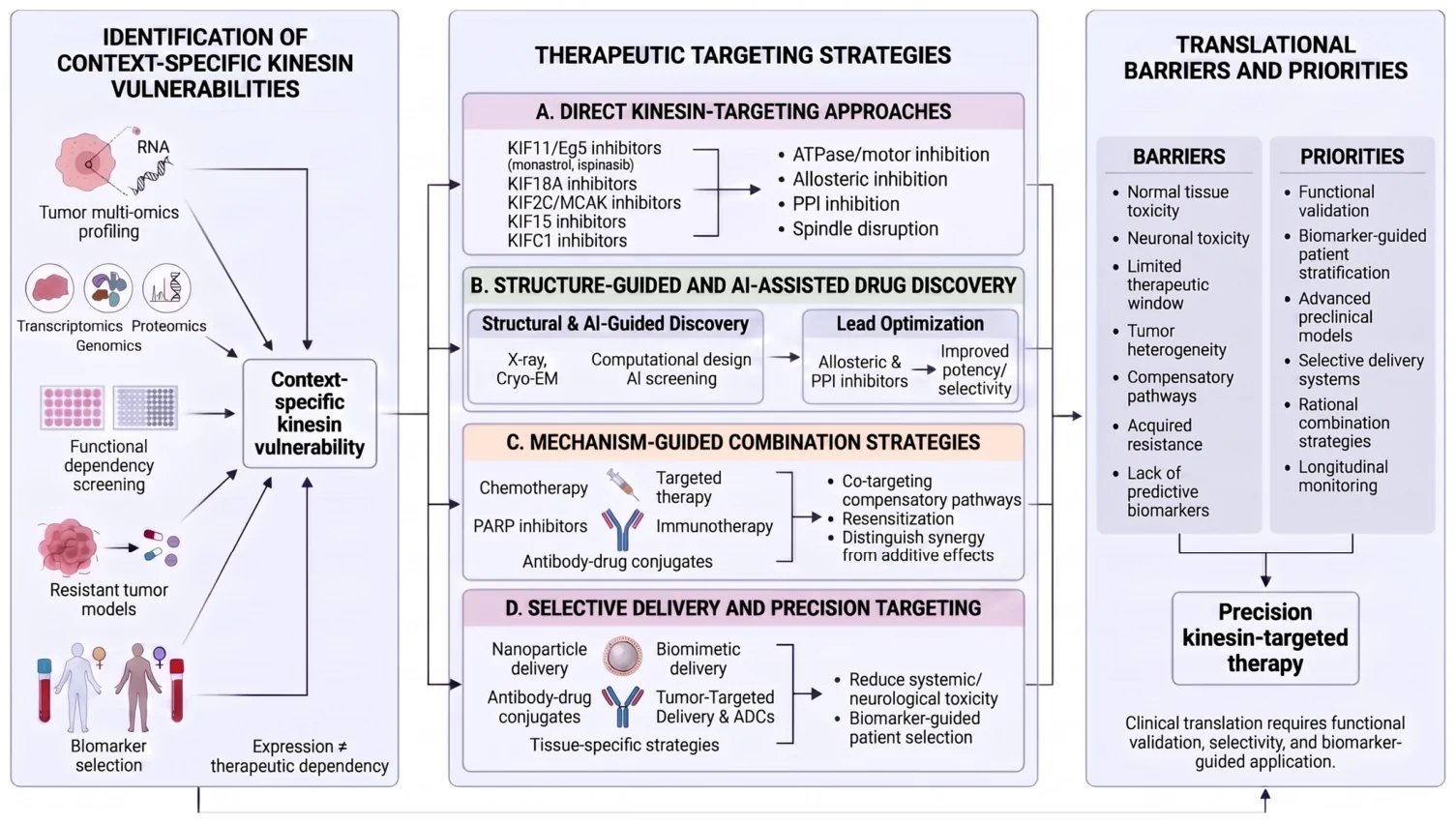
Therapeutic targeting strategies and translational challenges of kinesin inhibition in cancer. Current approaches include direct kinesin inhibitors, structure-guided drug discovery, combination strategies, and selective delivery approaches. Major translational barriers and future priorities for developing precision-oriented kinesin-targeted therapies are summarized. Different colors indicate distinct therapeutic strategy categories and translational components, while arrows represent the proposed relationships and workflow between different elements. Created in BioRender. Li, M. (2026) https://BioRender.com/b8q3ubz (accessed on 23 August 2026).

Importantly, evidence supporting kinesin targeting exists at multiple levels, including biochemical inhibition of motor activity, cellular inhibition of proliferation, reversal of resistance phenotypes, and clinical efficacy. These levels should be distinguished because pharmacological inhibition of a kinesin does not necessarily translate into selective antitumor activity or durable therapeutic benefit. Current kinesin-targeted strategies and major translational challenges are summarized in Figure 2.

### 4.1. Direct Kinesin Inhibitors: Natural Products, Natural-Product-Inspired Scaffolds, and Synthetic Chemical Probes

Direct kinesin inhibitors have provided important mechanistic evidence that motor activity can be pharmacologically manipulated. However, these compounds represent chemically diverse categories, including genuine natural products, natural-product-inspired molecules, and synthetic small-molecule inhibitors, which should be distinguished according to their origin and mechanism of action. The development of resistance to classic microtubule-targeting agents such as paclitaxel, a microtubule stabilizer that was originally derived from the bark of the Pacific yew tree, is frequently associated with the aberrant expression or function of kinesin family members involved in spindle dynamics [52,78]. These findings underscore the critical role of kinesin motors in mitotic fidelity and suggests their potential as therapeutic targets for overcoming drug resistance.

Among the earliest and most widely studied kinesin inhibitors is monastrol, which provided the first demonstration that a small molecule could selectively interfere with mitotic kinesin function [79]. However, monastrol should not be classified as a natural-product-derived compound. Instead, monastrol is a synthetic dihydropyrimidinone derivative and a selective allosteric inhibitor of the mitotic kinesin *Eg5*/*KIF11*. Structural and biochemical studies demonstrated that monastrol binds to an allosteric pocket within the Eg5 motor domain rather than competing directly with ATP. This interaction alters the mechanochemical cycle of Eg5, including microtubule-stimulated ADP release, thereby inhibiting microtubule sliding activity and preventing bipolar spindle formation [80].

Although monastrol established the feasibility of Eg5 inhibition, its relatively modest potency and pharmacological properties limited direct clinical translation. These limitations stimulated the development of chemically optimized Eg5 inhibitors and compounds targeting alternative allosteric regions of the motor domain. For example, cryo-electron microscopy studies revealed that GSK-1 inhibits human kinesin-5 through an unconventional allosteric mechanism by promoting a tight microtubule-bound state and trapping the motor in an ATP-like conformation. This finding demonstrated that kinesins can be targeted through multiple regulatory states beyond the classical monastrol-binding mechanism [81].

Natural products remain valuable sources of kinesin-targeting chemical probes. Tonantzitlolone A, a diterpene isolated from Stillingia sanguinolenta, induces monoastral spindle formation and interferes with kinesin-5–microtubule interactions. The synthetic enantiomer retains inhibitory activity, suggesting that natural-product-derived scaffolds can provide alternative approaches for modulating kinesin motor activity [82]. Similarly, adociasulfates isolated from marine sponges represent another class of natural-product kinesin inhibitors. Unlike monastrol, which acts through an allosteric motor-domain mechanism, adociasulfates inhibit kinesins through competitive interference with microtubule binding and display activity against multiple kinesin family members [83,84]. However, broad kinesin inhibition may limit therapeutic applicability because nonselective disruption of multiple motor proteins could increase toxicity and reduce tumor selectivity. In addition, whether these compounds retain activity in multidrug-resistant tumors with defined *ABCB1*/P-glycoprotein overexpression remains insufficiently established. Therefore, their ability to overcome transporter-mediated drug resistance should not be assumed without compound-specific validation.

Collectively, direct kinesin inhibitors provide valuable proof of concept that motor activity can be therapeutically manipulated. However, future development requires not only stronger inhibition but also improved selectivity, resistance-model validation, and demonstration of a clinically meaningful therapeutic window.

### 4.2. Research Progress on Mitotic Kinesin Inhibitors: Lessons from KIF11/Eg5 Clinical Development

The development of small-molecule inhibitors targeting mitotic kinesins, especially *KIF11* (Eg5), represents one of the most advanced strategies for overcoming therapeutic resistance in cancer. *KIF11* inhibitors such as ispinesib and filanesib (ARRY-520) have entered clinical trials for hematological malignancies and solid tumors, either as monotherapies or in combination with taxanes and platinum-based drugs. These agents primarily function by inhibiting the ATPase activity of Eg5 by disrupting centrosome separation and bipolar spindle formation, thereby inducing monopolar spindle assembly, prolonged mitotic arrest, mitotic catastrophe, and subsequent apoptotic cell death. Clinical studies demonstrated that *KIF11* inhibition is biologically active; however, clinical efficacy has generally been modest, particularly in solid tumors. Phase II studies of ispinesib in melanoma, prostate cancer, head and neck cancer, and hepatocellular carcinoma showed limited antitumor activity, highlighting the challenge of translating mitotic inhibition into durable clinical benefit [85,86,87]. Moreover, pharmacokinetic-pharmacodynamic analyses identified myelosuppression, particularly neutropenia, as an important dose-limiting toxicity of ispinesib, emphasizing the narrow therapeutic window associated with systemic inhibition of essential mitotic motors [88].

The clinical experience with *KIF11* inhibitors illustrates an important principle: target engagement and mitotic disruption alone are insufficient predictors of therapeutic success. Tumors may activate compensatory mechanisms, tolerate mitotic stress, or rely on alternative spindle-regulatory pathways. A major mechanism of resistance involves compensatory upregulation of *KIF15*, which can partially rescue spindle bipolarity in the absence of *KIF11* activity [89,90]. *KIF15*-mediated spindle maintenance represents one of the clearest examples of adaptive resistance to kinesin inhibition. When *KIF11* activity is impaired, tumor cells may become increasingly dependent on *KIF15*-mediated spindle assembly, creating both a resistance mechanism and a potential combination-treatment vulnerability [91].

The limited clinical success of *KIF11* inhibitors has also stimulated interest in alternative strategies, including tumor-selective kinesin dependencies, allosteric inhibitor design, and biomarker-guided patient selection. Rather than broadly inhibiting essential mitotic motors, future approaches may focus on identifying tumor states in which specific kinesins become disproportionately required for survival.

### 4.3. Development of Inhibitors Targeting Other Kinesin Members

Beyond *KIF11*, increasing evidence suggests that other kinesin family members may provide opportunities for more selective therapeutic targeting.

#### 4.3.1. *KIF18A*: Exploiting Chromosomal Instability-Associated Vulnerability

*KIF18A* regulates chromosome congression, kinetochore–microtubule dynamics, and mitotic fidelity. Recent studies have moved beyond expression-based associations and demonstrated that *KIF18A* represents a selective vulnerability in chromosomally unstable (CIN-high) tumors. Small-molecule inhibition of *KIF18A* preferentially impairs CIN-high cancer cells while producing weaker effects in genomically stable cells, providing a potential strategy to improve therapeutic selectivity compared with conventional antimitotic approaches [92]. Furthermore, selective *KIF18A* inhibitors, including VLS-1488, have entered early clinical evaluation, representing one of the first examples of a precision-oriented kinesin-targeting strategy based on tumor-specific dependency rather than simple overexpression [93].

#### 4.3.2. *KIFC1*/HSET: Targeting Centrosome Amplification Dependency

*KIFC1*/HSET, a minus-end-directed kinesin, is essential for survival of tumor cells with centrosome amplification. In these cells, *KIFC1* clusters supernumerary centrosomes and prevents lethal multipolar mitosis, whereas most normal cells are less dependent on this pathway. Chemical inhibitors targeting *KIFC1*, including AZ82 and CW069, have provided proof of concept that centrosome-amplified tumors may be selectively vulnerable to *KIFC1* inhibition. AZ82 disrupts *KIFC1*-dependent centrosome clustering, whereas CW069 induces apoptosis and restores docetaxel sensitivity in resistant prostate cancer models [94,95].

These findings are particularly relevant to drug resistance because they demonstrate that kinesin inhibition can be used not only to suppress proliferation but also to reverse established therapy-resistant phenotypes.

#### 4.3.3. Cargo-Transporting Kinesins: Challenges in Achieving Selective Inhibition

In contrast to mitotic kinesins, the therapeutic development of cargo-transporting kinesins remains substantially less advanced.

Cargo-transporting kinesins, including *KIF5B*/kinesin-1 family members, participate in essential physiological processes such as long-range intracellular transport, organelle positioning, and neuronal cargo trafficking. Therefore, systemic inhibition of these motors may produce a narrower therapeutic window than tumor-selective mitotic kinesins.

*KIF5B* is a component of the kinesin-1 motor complex and is required for microtubule-dependent transport of diverse intracellular cargos. Studies in neuronal systems have demonstrated that kinesin-1 dysfunction disrupts axonal transport and contributes to neurological disease phenotypes, highlighting the potential safety concerns associated with systemic inhibition of this motor family [96,97]. Accordingly, future *KIF5B*-directed therapeutic strategies may require approaches that achieve spatial or cellular selectivity rather than simple systemic motor inhibition. Potential strategies include tumor-targeted nanoparticles, receptor-directed delivery systems, or selective delivery of intracellular inhibitory payloads such as small molecules or nucleic-acid therapeutics. Importantly, *KIF5B* itself is an intracellular protein and therefore cannot serve as a conventional cell-surface antigen for direct antibody–drug conjugate targeting. Instead, future delivery approaches would require tumor-associated surface markers to transport *KIF5B*-directed payloads selectively into malignant cells. Thus, kinesin targeting strategies should consider the biological context of each motor: *KIF18A* may provide CIN-dependent selectivity, *KIFC1* may exploit centrosome amplification, whereas cargo motors such as *KIF5B* will likely require delivery-based solutions to overcome physiological constraints.

### 4.4. Resistance Challenges Faced by Synthetic Kinesin Inhibitors

Similar to other targeted therapies, kinesin inhibitors may encounter multiple resistance mechanisms.

#### 4.4.1. Target Alteration and Adaptive Motor Rewiring

One major mechanism involves the genetic alteration of the target protein, particularly mutations in the ATP-binding pocket of kinesin motors such as *KIF11*, which reduce inhibitor binding affinity and drug efficacy [98]. However, resistance mechanisms depend on the specific inhibitor-binding mode. For allosteric *KIF11* inhibitors such as monastrol derivatives, resistance-associated alterations may occur within or near allosteric regulatory regions rather than the conserved ATP-binding site. Structural understanding of inhibitor-binding regions is therefore essential for designing next-generation compounds capable of overcoming resistance.

A second major mechanism involves adaptive rewiring of kinesin networks. The best-characterized example is *KIF15*-mediated compensation after *KIF11* inhibition. When Eg5 activity is suppressed, tumor cells can maintain spindle bipolarity through *KIF15*-dependent mechanisms, allowing continued mitotic progression despite pharmacological inhibition of *KIF11* [98]. This phenomenon illustrates an important principle of kinesin-targeted therapy: resistance may not arise from loss of drug binding alone but from functional redundancy within the kinesin network.

#### 4.4.2. Drug Efflux and Multidrug Resistance

ATP-binding cassette transporters, particularly P-glycoprotein (P-gp/*ABCB1*), represent an established mechanism of multidrug resistance by reducing intracellular exposure to multiple anticancer agents. However, whether individual kinesin inhibitors are substrates of *ABCB1* requires compound-specific investigation. For example, the presence of *ABCB1* overexpression in resistant tumors does not automatically indicate that monastrol, adociasulfates, ispinesib, or other kinesin inhibitors will fail. Future studies should directly evaluate intracellular drug accumulation, transporter dependency, and activity in genetically defined *ABCB1*-overexpressing models [99]. Therefore, the ability of kinesin inhibitors to overcome transporter-mediated resistance should be demonstrated experimentally rather than inferred from general multidrug-resistance mechanisms.

#### 4.4.3. Tumor Heterogeneity and Therapeutic Window Limitations

Tumor heterogeneity represents another major challenge because kinesin dependency is unlikely to be uniform across all malignant cells. Molecular states such as chromosomal instability, centrosome amplification, lineage plasticity, and acquired drug-resistant phenotypes may determine whether a tumor becomes dependent on a specific kinesin. This concept explains why expression-based biomarker strategies alone may be insufficient. A highly expressed kinesin does not necessarily represent a therapeutic vulnerability unless the tumor demonstrates functional dependence on that motor.

A further challenge is the therapeutic window. Many kinesins are essential for normal cellular physiology, and systemic inhibition may affect normal proliferative tissues or specialized cell types. Clinical experience with *KIF11* inhibitors demonstrates that hematologic toxicity, particularly myelosuppression, can limit dosing intensity. Conversely, emerging strategies targeting *KIF18A* or *KIFC1* attempt to exploit tumor-specific dependencies, such as CIN or centrosome amplification, rather than relying solely on differential expression.

Taken together, these observations indicate that the development of kinesin inhibitors cannot be separated from the broader challenge of anticipating and suppressing compensatory mechanisms, thereby reinforcing the need for biomarker-guided and mechanism-based therapeutic strategies.

### 4.5. Mechanism-Guided Combination Strategies to Increase Efficacy and Reduce Resistance

Because tumor cells readily adapt to single-agent treatments, combination therapies are widely regarded as important strategies for maximizing the antitumor effects of kinesin-targeted drugs. However, rational combination therapy should not simply aim to increase cytotoxicity. The most informative combinations are those that directly address a known resistance mechanism, such as compensatory motor activation, mitotic adaptation, or survival pathway rewiring.

#### 4.5.1. *KIF11* Inhibition Combined with Mitotic Pathway Targeting

More broadly, preclinical studies suggest that the efficacy of *KIF11* inhibitors can be enhanced by co-targeting parallel mitotic pathways. For example, Aurora A inhibitors synergize with the *KIF11* inhibitor SB743921 by promoting monoastral spindle formation and mitotic catastrophe, and this combination was also shown to overcome *KIF15*-dependent SB743921 resistance. These findings suggest that rational co-targeting of mitotic regulators may improve *KIF11*-directed therapy and reduce compensatory resistance [91]. This study provides a mechanistically supported example in which combination therapy directly targets an adaptive resistance pathway rather than producing nonspecific additive toxicity. Similarly, genetic suppression of *KIF15* increases sensitivity to *KIF11* inhibitors, indicating that simultaneous targeting of complementary mitotic kinesins may increase cytotoxicity in selected tumor contexts [100]. Nevertheless, dual inhibition of essential mitotic motors may also increase toxicity. The same biological redundancy that provides a rationale for *KIF11*/*KIF15* co-targeting may narrow the therapeutic window in normal proliferating tissues. Therefore, clinical translation will require careful evaluation of dosing sequence, intermittent scheduling, and pharmacodynamic monitoring.

#### 4.5.2. Beyond Free-Drug Combinations: Targeted Delivery Strategies

An emerging direction is the integration of kinesin inhibitors with tumor-selective delivery systems. Nanoparticle-based, biomimetic, and stimulus-responsive platforms have been developed to improve intratumoral drug accumulation and reduce systemic exposure [101,102]. In addition, multimodal nanoplatforms that combine tumor-responsive delivery with tumor-microenvironment remodeling have shown the potential to enhance therapeutic efficacy in cancer models, providing a broader proof of concept for mechanism-guided delivery strategies [103]. Although these approaches have not yet demonstrated kinesin-specific therapeutic efficacy, they provide conceptual platforms for improving the tumor-selective delivery of intracellular therapeutic payloads. Such approaches may be particularly relevant for cargo-transporting kinesins, for which essential physiological functions may limit the feasibility of systemic inhibition. Overall, targeted delivery represents a promising strategy for improving the therapeutic window of kinesin-directed interventions, but direct validation in kinesin-dependent and resistance-relevant models remains necessary.

Future kinesin-targeted therapies will likely require three integrated components: (1) identification of a tumor-specific kinesin dependency, (2) selection of patients using predictive biomarkers rather than expression alone, and (3) pharmacological strategies or delivery systems that maximize tumor selectivity while minimizing toxicity.

## 5. Design and Optimization Strategies for Kinesin Inhibitors

### 5.1. Lead Scaffold Optimization and Mechanism-Guided Development of Kinesin Inhibitors

Small molecules targeting kinesins have evolved from initial phenotype-based discoveries toward mechanism-guided and structure-informed optimization strategies. The development of clinically relevant kinesin inhibitors requires not only identification of active compounds but also systematic refinement of their potency, selectivity, pharmacokinetic properties, and activity in resistance-relevant models.

A representative milestone in kinesin inhibitor development is monastrol, a synthetic dihydropyrimidinone derivative identified through phenotype-based chemical screening. Monastrol induced monopolar spindle formation by selectively inhibiting the mitotic kinesin Eg5 (*KIF11*), establishing kinesins as pharmacologically tractable targets distinct from conventional microtubule-targeting agents [79]. Subsequent biochemical studies demonstrated that monastrol does not inhibit Eg5 through direct competition with ATP binding. Instead, monastrol functions as an allosteric inhibitor by preferentially binding the Eg5–ADP state and interfering with conformational transitions required for productive microtubule interaction and motor activity [80]. These findings established allosteric regulation of kinesin conformational dynamics as an important principle for inhibitor development.

Although monastrol provided a proof-of-concept for kinesin inhibition, its limited potency and drug-like properties stimulated extensive medicinal chemistry optimization. Modification of the dihydropyrimidinone scaffold generated C5-substituted fatty dihydropyrimidinones (DHPMs), among which compound 10c showed micromolar antiproliferative activity against breast and gastric cancer cells with improved selectivity toward normal cells [104]. Similarly, green synthetic approaches produced DHPM derivatives such as compound C9, which exhibited mixed-type Eg5 ATPase inhibition and cytotoxic activity, further supporting the feasibility of scaffold optimization for kinesin-directed therapy [105].

From a medicinal chemistry perspective, successful kinesin inhibitor development requires optimization beyond intrinsic activity. Key parameters include target selectivity, cellular permeability, metabolic stability, and efficacy under clinically relevant resistance conditions. Therefore, future optimization strategies should incorporate resistance-associated molecular alterations and tumor-specific kinesin dependencies rather than focusing solely on biochemical inhibition.

Furthermore, diversification of chemical scaffolds has expanded the available chemical space for Eg5 inhibition. Morphology-guided profiling approaches have identified spirooxindole-isooxindoles as a distinct Eg5 inhibitor chemotype, demonstrating that chemically diverse scaffolds can be exploited for kinesin-targeted drug development [106].

Structural studies have further enhanced the understanding of kinesin inhibitor optimization. Cryo-electron microscopy analysis of human kinesin-5 bound to GSK-1 revealed that inhibitor binding at regulatory regions of the motor domain can stabilize specific conformational states and restrict nucleotide-dependent mechanochemical transitions, providing a structural basis for designing next-generation kinesin inhibitors [81,107].

### 5.2. Structural and Computational Strategies for Kinesin Inhibitor Design

Structural biology provides a fundamental framework for rational drug design by resolving the atomic architectures of kinesin proteins and their interactions with ligands. High-resolution techniques such as X-ray crystallography and cryo-electron microscopy (cryo-EM) have enabled detailed characterization of kinesin motor domains, microtubule interactions, and inhibitor-binding conformations [108,109,110,111].

These structural insights reveal not only canonical ATP-binding pockets but also alternative druggable sites, including allosteric pockets and protein–protein interaction (PPI) interfaces. Unlike ATP-competitive strategies, targeting allosteric regions may provide opportunities to achieve improved selectivity by exploiting kinesin-specific conformational states. Computational fragment-based approaches have further explored hydrophobic sub-pockets within Eg5 allosteric binding regions, providing additional strategies for rational inhibitor optimization.

Structure-based virtual screening has also expanded the discovery of novel kinesin inhibitors. By screening approximately 500,000 compounds against predicted Eg5 allosteric pockets, Zhang et al. identified SRI35566 as a novel Eg5 inhibitor. SRI35566 directly interacted with Eg5, induced monopolar spindle formation, and suppressed cancer cell proliferation, demonstrating the feasibility of targeting alternative kinesin regulatory sites through computational approaches [112].

Consistent with this strategy, molecular dynamics, pharmacophore modeling, and 3D-QSAR studies of biphenyl-type Eg5 inhibitors have shown that stabilization of the flexible L11 region contributes to productive engagement of the α4/α6 allosteric pocket, thereby informing the optimization of this inhibitor class [113]. Furthermore, structural analysis of kinesin complexes with endogenous binding partners enables identification of additional therapeutic opportunities. Protein–protein interaction interfaces may serve as alternative targets for disrupting kinesin-associated oncogenic functions, as demonstrated by structural characterization of kinesin-associated regulatory complexes [114,115].

Beyond Eg5, recent studies suggest that selective targeting of other kinesin family members may provide new therapeutic opportunities. Small-molecule inhibition of *KIF18A* revealed a selective vulnerability in chromosomally unstable cancers, particularly *TP53*-mutant breast and ovarian cancer models. These findings indicate that kinesin inhibitors may achieve improved therapeutic windows by exploiting tumor-specific genetic and cellular dependencies rather than broadly targeting mitotic processes [92].

In conjunction with experimental structural biology, AI-assisted and virtual screening approaches have been applied to identify kinesin inhibitors. For example, a generative LSTM workflow combined with docking, molecular dynamics simulations, and MM-GBSA prioritization yielded novel *KIF11*-binding candidates with favorable predicted properties [116]. In addition, shape- and similarity-based virtual screening led to the identification of YL001, a potent Eg5 inhibitor that retained activity in Taxol-resistant ovarian and 6TG-resistant breast cancer models and showed antitumor efficacy in vivo [117].

Emerging artificial intelligence-based drug–target interaction prediction models, such as deep-learning frameworks based on multiscale drug and target features, may facilitate the prioritization of candidate kinesin–inhibitor interactions [118]. However, computational prediction alone cannot determine whether a kinesin represents a genuine therapeutic dependency. Integration with functional genomic screening, transcriptomic and proteomic profiling, and drug-response datasets will be necessary to distinguish actionable kinesin vulnerabilities from passenger associations.

Taken together, structure-guided optimization and computational strategies have expanded the chemical and mechanistic space available for kinesin inhibitor development. However, most kinesin inhibitors remain at the preclinical stage, and further validation in clinically relevant models of drug resistance is required to determine their translational potential.

## 6. Expression- and Genomic-Based Biomarker Potential of Kinesin-Related Drug Resistance

The expression levels of specific kinesin family (KIF) genes, such as *KIF4A* and *KIF20A*, in tumor tissue, as measured by mRNA or protein quantification, may serve as useful biomarkers for predicting tumor aggressiveness and identifying tumor contexts characterized by increased proliferative activity, mitotic dependency, or aggressive biological behavior. This concept is grounded in the broader principle that tumor molecular profiles can shape treatment responses. For example, in addition to conventional genomic markers, transcriptional profiling has been shown to improve patient stratification and predict therapeutic sensitivity in several tumor settings. Similarly, proteomic and transcriptomic features have been associated with drug sensitivity or resistance in hematologic and solid malignancies [119,120,121,122]. In the context of kinesin biology, these findings suggest that aberrant expression of mitosis-related genes, including KIF members, may provide clinically relevant information for predicting responsiveness to therapies that disrupt spindle function or microtubule-dependent processes. However, many reported kinesin expression signatures currently represent prognostic associations reflecting tumor proliferation, disease progression, or aggressive biological states rather than validated predictive biomarkers of treatment response. Importantly, prognostic biomarker capability should not be directly interpreted as evidence of therapeutic sensitivity or resistance prediction. However, compared with general expression-based predictive models in oncology, direct validation of individual kinesin genes as predictive biomarkers of chemotherapy response remains limited, and further tumor-specific studies are needed.

In addition, somatic mutations and copy number variations (CNVs) in kinesin genes may influence treatment responses by altering kinesin functions or expression levels; thus, genomic analysis is a potentially informative complementary approach to expression-based assessment. More broadly, the genomic landscape of a tumor influences therapeutic outcomes, as revealed by studies on driver mutations, copy number alterations, and co-mutation patterns across multiple cancer types [123,124,125,126]. This principle may also apply to kinesin-related vulnerabilities. For example, in pancreatic cancer, multiomics analyses revealed that *KIF20B* copy number variations are associated with immune infiltration patterns and that *KIF20B* has prognostic value, indicating that kinesin-related genomic alterations may be relevant biomarkers in selected tumor contexts [127]. Nevertheless, compared with the evidence supporting expression-based associations, direct evidence establishing kinesin gene mutations or CNVs as predictive biomarkers of drug resistance is lacking. Therefore, although the systematic genomic sequencing of kinesin genes may be informative, its clinical utility must be validated in defined tumor contexts and treatment settings. Future biomarker development will likely require integration of kinesin expression patterns with genomic alterations, functional dependency screening, and treatment-response datasets. Such multi-dimensional approaches may help distinguish kinesin alterations that merely reflect tumor biology from those that represent actionable therapeutic vulnerabilities.

## 7. Application of Preclinical Models in the Study of Kinesins and Drug Resistance

### 7.1. Gene-Edited Cell Lines and Organoid Models

Gene-edited cellular models can be used to directly establish causal links between kinesin dysregulation and therapy-related phenotypes. By using CRISPR-Cas9 to knock out, edit, or modulate specific KIF genes in isogenic systems, researchers can determine how individual kinesins influence mitotic fidelity, cell survival, and drug response, thereby moving beyond correlative analyses.

Importantly, genetic perturbation approaches provide critical evidence for distinguishing whether kinesin alterations represent functional drivers of therapeutic resistance rather than merely reflecting aggressive tumor states. Combining gene knockout or knockdown strategies with drug-response assays, rescue experiments, and orthogonal validation approaches can help define kinesin-dependent vulnerabilities with greater mechanistic confidence.

A representative example is the generation of CENP-E-knockout HeLa cells by CRISPR/Cas9, which directly revealed the functions of this kinetochore-associated kinesin. The loss of CENP-E resulted in chromosome misalignment, aberrant BubR1 localization, and marked mitotic defects, and this model was also used to validate the specificity and cellular effects of CENP-E-targeted inhibitors, supporting its utility for the development of kinesin-focused anticancer drugs [128]. In addition, combined CRISPR/Cas9 functional genomic screening and tumor genomic analysis revealed that *KIFC1*/HSET is involved with centrosome amplification in lung adenocarcinoma. *KIFC1* loss impaired centrosome clustering and revealed a lethal mitotic vulnerability, highlighting how kinesin perturbations can reveal therapeutically actionable dependencies in chromosomally unstable tumors [129]. Gene-edited systems have also been used to link kinesins to chemoresistance. In bladder cancer, *TP53* knockout upregulated *KIFC1*, whereas *KIFC1* knockdown increased sensitivity to cisplatin. Consistent with these findings, *KIFC1* positivity in patient tumors is correlated with poor outcomes after cisplatin-based chemotherapy, supporting a functional connection between *KIFC1* expression and platinum resistance [26].

However, expression-based associations alone are insufficient to establish kinesins as universal resistance determinants. For example, *KIFC1*-mediated cisplatin tolerance requires functional validation in additional models because kinesin dependencies may vary according to tumor lineage, genomic background, and therapeutic context.

More recently, integrated genomic screening combined with patient-derived models has demonstrated how kinesin-associated mitotic regulators can reveal context-dependent therapeutic vulnerabilities. In pancreatic cancer, transcriptomic comparison of PARP inhibitor-sensitive and resistant patient-derived xenograft models identified *TPX2* as a determinant of olaparib response. Genetic inhibition of *TPX2* restored PARP inhibitor sensitivity, and this vulnerability was further validated in patient-derived organoid and xenograft models, supporting the concept that mitotic regulators associated with kinesin networks may represent synthetic lethal targets in resistant tumors [130].

Furthermore, organoid systems are valuable because they preserve three-dimensional architectures and phenotypes in tissue contexts. Although tumor organoid studies focused on kinesin-mediated drug resistance remain limited, organoid-based models provide useful frameworks for examining lineage-specific and spatial consequences of kinesin perturbations. For example, human iPSC-derived organoids with KIF26A loss-of-function exhibited defects in neuronal migration, localization, neurite development, and apoptosis, as well as transcriptional changes in the MAPK, *MYC*, and *E2F* pathways [131]. Similarly, in human iPSC-derived forebrain organoids, *KIF5C* deficiency disrupts neuronal migration, dendritic branching, and spine growth [132]. Although these studies were not performed in cancer models, they demonstrate that organoid platforms can capture tissue-specific consequences of kinesin perturbation and may provide future opportunities to evaluate tumor-specific kinesin dependencies, drug sensitivity, and resistance evolution in a more physiologically relevant environment.

### 7.2. Transgenic Mice and Patient-Derived Xenograft Models

Genetically engineered mouse models are essential for examining the in vivo consequences of kinesin dysregulation within intact tissue and microenvironmental contexts. Conditional or cell type-specific perturbation of kinesin genes can reveal intrinsic tumor and stromal functions that are difficult to capture in vitro. These models are particularly important because kinesins may regulate therapeutic responses not only through tumor-cell intrinsic mechanisms but also through effects on vascular remodeling, immune regulation, and tumor–stroma interactions. An illustrative example is the use of endothelial-specific Kif3a-knockout mice in ovarian cancer research. The loss of Kif3a, a gene required for primary cilium assembly, promoted the endothelial–mesenchymal transition, vascular abnormalities, and tumor-supportive remodeling of the ovarian microenvironment. Mechanistically, these effects were linked to *EphA2* activation after ciliary loss, indicating that in vivo kinesin perturbations can reveal stromal mechanisms relevant to tumor progression and potentially to treatment response [76].

Patient-derived xenograft (PDX) models represent complementary translational systems because the key histopathological and molecular characteristics of the original tumor are preserved. Compared with conventional xenograft systems, PDX models provide an intermediate level of evidence between mechanistic discovery and clinical translation because they allow evaluation of kinesin dependency in patient-derived tumor contexts. In soft tissue sarcoma, *KIFC1* was identified as both a prognostic biomarker and a therapeutic target, and *KIFC1* knockout inhibited tumor growth in vitro and in vivo while also suppressing the growth of PDX tumors, as revealed by an *FXR1*-*MAD2L1*-related senescence program [133].

PDX systems have also been applied to test kinesin-targeted therapies. The selective Eg5 inhibitor LY2523355 exhibits robust target-mediated antitumor activity in vivo, including complete responses in several xenograft and PDX models, and tumor phospho-histone H3 was proposed as a pharmacodynamic response marker [134]. Similarly, in pancreatic cancer, Eg5-positive PDX models respond to ispinesib, whereas Eg5 expression in patient tumors is associated with poor clinical outcomes, supporting the translational relevance of this mitotic kinesin as both a drug target and biomarker [135].

In addition to direct pharmacologic inhibition, kinesin-associated antigens can be exploited therapeutically. In hepatocellular carcinoma, *KIF20A* was validated as an immunogenic tumor-associated antigen, and a *KIF20A*-based thermosensitive hydrogel vaccine increased the efficacy of PD-L1 blockade in subcutaneous and orthotopic CDX models and immune-humanized PDX models, accompanied by improved dendritic cell maturation and increased CD8^+^ T-cell infiltration [75].

**Figure 3:**
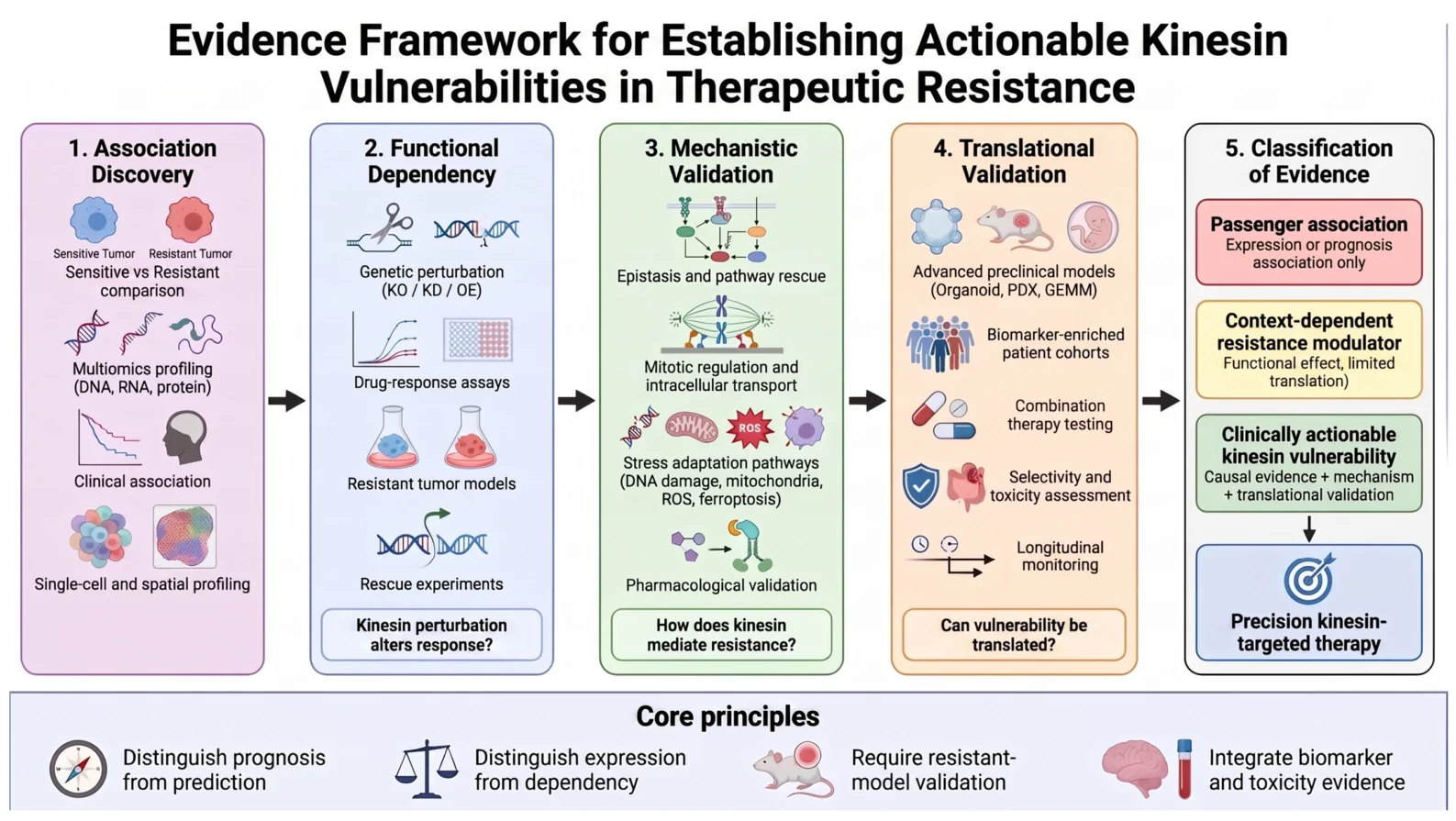
Evidence framework for evaluating kinesin vulnerabilities in therapeutic resistance. A stepwise framework is proposed to distinguish association-only findings from functionally validated and translationally relevant kinesin vulnerabilities by integrating functional dependency, mechanistic validation, translational assessment, and evidence classification. Different colors represent distinct evaluation stages and evidence categories, while arrows indicate the sequential workflow and relationships among the assessment steps. Created in BioRender. Li, M. (2026) https://BioRender.com/wwtsd4n (accessed on 23 August 2026).

Together, these findings emphasize that preclinical models should be selected according to the biological question being addressed. Gene-edited systems are most suitable for establishing causal relationships between kinesin alteration and resistance phenotypes, organoids for evaluating context-dependent drug responses, genetically engineered models for dissecting tumor–microenvironment interactions, and PDX systems for translational validation of kinesin-directed therapeutic strategies. Collectively, these approaches support a stepwise evidence framework for distinguishing association-only findings from functionally validated and clinically actionable kinesin vulnerabilities in therapeutic resistance (Figure 3).

## 8. Challenges and Translational Priorities

### 8.1. Current Challenges and Limitations

Despite the growing recognition of kinesins as regulators of drug resistance and promising therapeutic targets in cancer, several major challenges hinder their clinical translation. A central issue is target specificity. Many kinesin family members perform indispensable physiological functions in normal cells, particularly in neurons and actively proliferating tissues such as bone marrow and intestinal epithelium. Therefore, the development of highly selective inhibitors that suppress tumor-promoting kinesin activity without causing unacceptable toxicity remains a major obstacle. This challenge is especially relevant for broadly expressed kinesins involved in intracellular trafficking, as their systemic inhibition may disrupt essential homeostatic processes.

Furthermore, the high degree of conservation among kinesin motor domains creates additional challenges for isoform-selective drug development, as multiple family members share similar ATP-binding and microtubule-interacting regions. Therefore, targeting unique regulatory pockets, noncanonical domains, or tumor-specific dependency states may be required to achieve clinically meaningful selectivity.

A second major barrier is the evolution of acquired resistance. Tumor cells may not be affected by kinesin-targeted treatment owing to target mutation, the activation of bypass pathways, functional compensation by related kinesins, or phenotypic switching. Owing to the compensatory rewiring of signaling and trafficking networks, the inhibition of a single kinesin is often insufficient for durable tumor control.

A third limitation is the lack of robust predictive biomarkers. At present, there are no sufficiently validated biomarkers to reliably identify patient populations that are most likely to benefit from kinesin-targeted interventions. Given the heterogeneity in kinesin expression, subcellular localization, posttranslational regulation, and functional dependency across tumor types, biomarker-guided stratification is critical to increase the precision of therapeutic approaches.

In addition, kinesin biology is likely more complex than initial evaluations suggested. In addition to their canonical functions as microtubule-based motors, some kinesin-related proteins and motor-associated regulators may exhibit noncanonical activities in functions such as signal regulation, receptor trafficking, RNA control, and stress adaptation [136,137,138]. Dynamic posttranslational regulation and the complexity of the tumor microenvironment also introduce challenges in the development of relevant therapeutics; however, these factors remain insufficiently characterized in resistant tumors.

### 8.2. Future Research Directions

Future studies should prioritize the development of more selective and mechanistically sophisticated inhibitors. In particular, allosteric inhibitors and protein–protein interaction (PPI) inhibitors may offer greater selectivity than ATP-competitive compounds, especially for kinesins with highly conserved motor domains. Structural biology has already demonstrated the feasibility of identifying noncanonical pockets and conformationally restricted inhibitory regions, suggesting that next-generation kinesin inhibitors may increasingly rely on allosteric and interface-based designs [139,140,141].

Another important direction is the exploration of inhibition strategies involving two or more kinesins. Because tumor cells often exploit functional redundancy among kinesins, the simultaneous targeting of complementary motor kinesins, such as *KIF11* and *KIF15*, may provide a potential strategy to reduce compensatory adaptation and overcome resistance associated with kinesin network redundancy [100]. More broadly, compared with single-agent therapies, the rational co-targeting of kinesin dependency and downstream resistance pathways may result in stronger anti-resistance effects.

Beyond pharmacological inhibition, future studies should integrate functional genomic approaches with single-cell and multiomics analyses to identify context-specific kinesin dependencies. Such approaches may help distinguish essential resistance drivers from adaptive passenger alterations and define which tumor states are most likely to respond to kinesin-directed therapies.

Additionally, the understanding of noncanonical kinesin biology in resistant tumor settings must be improved. In particular, clarifying kinesin functions in the tumor microenvironment, metastatic dissemination, extracellular vesicle trafficking, and posttranslational regulation may reveal additional regulatory vulnerabilities [131,137,138].

Longitudinal studies combining genetic perturbation, spatial profiling, and treatment-response monitoring will be particularly important for understanding how kinesin dependencies evolve during therapy and metastatic progression.

### 8.3. Translational Priorities: Biomarker-Guided Combinations and Advanced Models

Future progress in kinesin-related drug resistance research will depend on integrating kinesin biology into more rigorous translational frameworks. Because resistant tumors exhibit substantial interpatient, intrapatient, and clonal heterogeneity, successful treatment will require repeated reassessment of tumor states through tissue biopsies, liquid biopsies, and longitudinal molecular profiling. Such strategies are essential for identifying evolving resistance pathways, selecting appropriate subsequent therapies, and designing biomarker-guided combination regimens [142,143,144]. Functional imaging and liquid biopsy approaches may provide complementary strategies for monitoring tumor evolution and treatment adaptation. However, kinesin-specific longitudinal biomarkers remain to be established, and their clinical application will require validation in prospective treatment cohorts [145,146,147].

Importantly, future therapeutic advances are likely to involve rational combination therapies rather than kinesin-targeted monotherapies. Depending on the tumor context, kinesin-directed agents may need to be combined with chemotherapy, radiotherapy, immunotherapy, PARP inhibitors, antibody-drug conjugates, or targeted kinase inhibitors to achieve more durable responses. Such combination strategies should be guided by mechanistic evidence rather than empirical drug combinations, with functional validation required to determine whether kinesin inhibition produces true synergistic effects or merely additive toxicity.

To evaluate such approaches more accurately, future studies must also incorporate advanced preclinical models that better recapitulate tumor complexity, including patient-derived organoids and patient-derived xenograft models. These systems will be particularly valuable for validating kinesin dependency, testing biomarker-guided strategies, and determining whether candidate therapies can delay or reverse resistance phenotypes.

## 9. Conclusions and Perspectives

Kinesin superfamily proteins are increasingly recognized as important contributors to drug resistance in cancer. Accumulating evidence supports the concept that specific kinesin family members contribute to therapy adaptation through regulation of mitotic progression, chromosome dynamics, DNA damage responses, intracellular trafficking, and stress-adaptive cell fate programs. These findings suggest that selected kinesins may represent mechanistic regulators of resistance and potential therapeutic vulnerabilities in specific tumor contexts. However, compared with the evidence for mitotic kinesins, the involvement of individual kinesins in processes such as drug efflux, immune evasion, and broader remodeling of the tumor microenvironment is lacking, and direct validation is needed.

From a translational perspective, the most important priorities are to define context-specific kinesin dependencies, distinguish functional resistance drivers from adaptive passenger alterations, develop robust predictive biomarkers, and test kinesin-directed strategies in advanced preclinical systems that better preserve tumor heterogeneity and resistance states. Overall, progress in this field will depend less on expanding descriptive associations and more on converting kinesin-related biology into testable, clinically actionable therapeutic frameworks.

## Figures and Tables

**Table 1:** Evidence landscape of kinesin-associated therapeutic resistance in cancer.

Kinesin	Resistance-Relevant Functional Class	Cancer Type	Therapy	Resistance Type	Proposed Mechanism	Experimental Model	Functional Evidence	Resistance Reversal	In Vivo/Clinical Evidence	Evidence Level	Ref.
*KIFC1*	Mitotic/centrosome-clustering	Bladder cancer	Cisplatin	Baseline sensitivity modulation; established cisplatin-resistant cell line not used	*KIFC1* depletion increases cisplatin sensitivity; reduced CD44 expression was proposed as a partial mechanistic link, but direct pathway rescue was not established.	5637 and UMUC3 bladder-cancer cells with *KIFC1* siRNA; IHC cohort of 46 patients treated with cisplatin-based chemotherapy.	*KIFC1* siRNA KD + cisplatin dose–response/IC50 testing + CD44 expression analysis; no *KIFC1* OE, rescue, or pharmacological target-engagement experiment.	Sensitization only; reversal of a pre-established cisplatin-resistant phenotype was not tested.	*KIFC1* positivity was associated with poorer survival after cisplatin-based chemotherapy, but not with objective cisplatin response (CR/PR vs. SD/PD); no in vivo validation.	C	[26]
*KIF11*	Mixed/multifunctional	Lung adenocarcinoma	Cisplatin	Acquired (DDP-resistant cells)	CaSR physically interacts with *KIF11*. *KIF11* suppression reduces *BRCA1*, cyclin B1 and *CDK1*-associated G2/M/DNA-repair signaling and increases cisplatin sensitivity in resistant LUAD cells.	A549-DDP and H1299-DDP cells generated by stepwise cisplatin exposure versus parental cells; CaSR/*KIF11* perturbation studies. An A549-DDP xenograft was used for the upstream CaSR-targeting arm.	*KIF11* siRNA + pharmacological *KIF11* inhibition (EG5 inhibitor V) + colony formation/cisplatin-sensitivity assays + pathway immunoblotting; no *KIF11* OE or molecular rescue.	Yes, in vitro. Genetic or pharmacological *KIF11* inhibition increased cisplatin sensitivity in established DDP-resistant LUAD cells.	No *KIF11*-specific in vivo or clinical resistance validation. The xenograft and chemotherapy-survival analyses primarily support the upstream CaSR arm.	B	[31]
*KIF2C*/MCAK	Mitotic/microtubule-regulatory	Triple-negative breast cancer	Paclitaxel and other MTAs	Acquired/selected chemoresistance with MTA cross-resistance	*KIF2C* preferentially depolymerizes polyglutamylated microtubules even in the presence of paclitaxel, thereby counteracting microtubule stabilization. The inhibitor 7S9 traps/inhibits *KIF2C* on microtubules and restores MTA sensitivity.	Human MDA-MB-231 and mouse 4T1 paclitaxel-resistant derivatives versus sensitive cells; resistant 4T1 clones; purified-protein/microtubule mechanistic assays; chemoresistant TNBC mouse model.	*KIF2C* shRNA + pharmacological inhibition (7S9) + biochemical/microtubule-depolymerization assays + tubulin-PTM mechanistic validation.	Yes. *KIF2C* depletion or 7S9 resensitized resistant cells to paclitaxel and 7S9 reduced cross-resistance to multiple clinically used MTAs.	Yes, in vivo: 7S9 + paclitaxel reduced growth of chemoresistant TNBC tumors in mice. No clinical treatment-response validation.	A	[32]
*KIF2C*/MCAK	Mitotic/microtubule-regulatory	CHO cell model (non-human; not tumor-specific)	Paclitaxel; epothilone A	Experimentally induced by gain of function	MCAK overexpression increases microtubule detachment from centrosomes and reduces microtubule polymer, opposing microtubule-stabilizing drugs.	Tet-off FLAG-MCAK CHO cells; stable clones and a paclitaxel-selected FLAG-MCAK-expressing population.	Inducible MCAK OE + conditional suppression of ectopic MCAK with tetracycline + microtubule polymer/detachment assays.	Yes in the induced-resistance model. Paclitaxel resistance required ectopic MCAK expression and was lost when MCAK expression was repressed.	No in vivo or clinical evidence; the model is non-tumor CHO cells.	B	[33]
*KIF14*	Mixed/multifunctional	Advanced prostate cancer	Docetaxel; cabazitaxel	Acquired docetaxel resistance with cabazitaxel cross-resistance	*KIF14* is upregulated in docetaxel-resistant cells and maintains *AKT* phosphorylation. *KIF14* silencing lowers *p-AKT* and restores taxane sensitivity; *AKT* inhibition attenuates the additional resistance-reversal effect of *KIF14* silencing, supporting a *KIF14*-*AKT* relationship.	DU145/DTX50 and PC-3/DTX30 docetaxel-resistant prostate-cancer cells versus parental DU145 and PC-3 cells; cabazitaxel cross-resistance testing.	*KIF14* siRNA + docetaxel/cabazitaxel drug-response, proliferation, clonogenic and apoptosis assays + *AKT*-pathway epistasis with MK-2206; no *KIF14* OE/rescue.	Yes, in vitro. *KIF14* silencing resensitized established docetaxel-resistant cells to both docetaxel and cabazitaxel.	No in vivo or clinical resistance-validation reported.	B	[34]
*KIF26B*	Mixed/multifunctional	Ovarian cancer	Paclitaxel	Acquired/established paclitaxel resistance	*KIF26B* supports the paclitaxel-resistant phenotype by limiting paclitaxel-induced microtubule stabilization, cell-cycle arrest and apoptosis. *SLC7A11* is functionally linked to this response: *SLC7A11* knockdown partially reverses the paclitaxel sensitization caused by *KIF26B* depletion, although a direct physical interaction remains unvalidated.	HeyA8 parental cells and HeyA8-R cells generated by 13 months of stepwise paclitaxel exposure (resistance index about 5.4); retrospective ovarian-cancer chemotherapy-response datasets.	Stable *KIF26B* shRNA + paclitaxel dose–response/IC50 + clonogenic assays + microtubule immunofluorescence + cell-cycle/apoptosis assays + *KIF26B*/*SLC7A11* double-knockdown epistasis.	Yes, in vitro. *KIF26B* knockdown reduced paclitaxel resistance in established HeyA8-R cells; *SLC7A11* co-depletion partially restored the resistant phenotype.	Retrospective database association between *KIF26B* expression and chemotherapy resistance across ovarian-cancer treatment subsets; no in vivo validation or prospective clinical response validation.	B	[35]
*KIF23*	Mixed/multifunctional	Chronic myeloid leukemia	Imatinib	Acquired imatinib resistance	*KIF23* activates Wnt/β-catenin signaling and maintains protective autophagy in imatinib-resistant cells. Pharmacological reactivation of autophagy or Wnt/β-catenin reverses the chemosensitizing effect of *KIF23* depletion.	K562 parental cells and acquired imatinib-resistant K562/R cells; serum samples from imatinib-sensitive and imatinib-resistant CML patients.	*KIF23* KD + direct imatinib IC50/proliferation/apoptosis assays + autophagy assays. Rapamycin partially rescued the *KIF23*-KD phenotype, and the Wnt activator BML-284 restored autophagy/survival and increased imatinib IC50 after *KIF23* depletion.	Yes. *KIF23* knockdown reduced imatinib IC50 in K562/R cells and increased apoptosis; pathway reactivation partially restored resistance.	No in vivo validation. Clinical association: *KIF23* mRNA/protein was higher in serum from imatinib-resistant than imatinib-sensitive CML patients; no prospective treatment-response validation.	B	[36]
*KIF4A*	Mixed/multifunctional	Non-small-cell lung cancer	Cisplatin	Treatment-induced tolerance/sensitivity modulation; established resistant line not used	*KIF4A* supports DNA double-strand-break repair through *BRCA2*/*RAD51* focus formation and *PARP1* activity; depletion increases cisplatin-induced damage and arrest.	H1299 human NSCLC cells treated with cisplatin.	siRNA KD + DDR/cell-cycle assays.	Sensitization only; reversal of an established resistant phenotype was not tested.	No in vivo or clinical resistance-validation.	C	[37]
*KIF11*	Mixed/multifunctional	Colorectal cancer	Oxaliplatin	Baseline/intrinsic sensitivity modulation; established resistant model not used	*KIF11* depletion enhances DNA damage and apoptosis through p53 activation and GSK3beta deactivation.	HT29 and HCT116 CRC cells; CRC tissues; xenograft used to assess *KIF11*-dependent tumor growth.	*KIF11* KD + p53-pathway pharmacological rescue.	Sensitization only; oxaliplatin-resistant phenotype was not pre-established.	*KIF11* KD suppresses tumor growth in vivo; oxaliplatin-resistance reversal in vivo not established.	B	[38]
*KIF11*	Mitotic/microtubule-regulatory with stemness/cell-cycle effects	Glioma	5-fluorouracil (5-FU)	Experimentally induced gain-of-function resistance/baseline sensitivity modulation; no established resistant derivative	*KIF11* promotes stem-like properties and cell-cycle progression, increasing *KLF4*/*NANOG*/*OCT4*/*LIN28*/CD133 and cyclin-associated programs. These phenotypes accompany increased 5-FU resistance, although a downstream resistance-specific rescue was not performed.	U87 cells with *KIF11* overexpression and U251 cells with *KIF11* knockdown; TCGA analyses and paired glioma/normal clinical tissues.	Gain- and loss-of-function: *KIF11* OE increased 5-FU resistance in U87 cells, whereas *KIF11* KD sensitized U251 cells to 5-FU; stemness, sphere/soft-agar, proliferation and cell-cycle assays were also performed.	Sensitization/induced resistance demonstrated, but not reversal of a pre-established resistant phenotype; no molecular rescue experiment.	Clinical expression/prognostic association only; no 5-FU treatment-response cohort and no in vivo drug-resistance validation.	B	[39]
*KIF4A*	Mixed/multifunctional	Colon cancer	Oxaliplatin	Acquired/established oxaliplatin resistance	*KIF4A* promotes glycolytic reprogramming and cancer stemness while suppressing oxidative phosphorylation, thereby facilitating oxaliplatin resistance. *KIF4A* overexpression-induced resistance and stemness are reversed by glycolysis inhibition.	HCT116-R oxaliplatin-resistant colon-cancer cells; *KIF4A* knockdown/overexpression models; lactate and Seahorse metabolic assays; oxaliplatin-resistant mouse xenograft.	*KIF4A* KD + OE + lactate/Seahorse metabolic profiling + glycolysis-inhibitor rescue.	Yes. *KIF4A* perturbation altered oxaliplatin response, and glycolysis inhibition reversed *KIF4A* overexpression-induced oxaliplatin resistance and stemness.	Oxaliplatin-resistant xenograft validation supported the resistance/stemness phenotype; no clinical treatment-response validation reported.	A	[40]
*KIF20A*	Mitotic/cytokinesis; EMT-associated	Glioma	Temozolomide (TMZ)	Computational/clinical response association; direct *KIF20A*-mediated TMZ resistance not established	*KIF20A* is associated with glioma malignancy and EMT and was linked to predicted/observed TMZ response. The study did not experimentally establish a *KIF20A* → TMZ-resistance mechanism.	TCGA/CGGA cohorts; GSE68029 containing TMZ-sensitive and TMZ-resistant glioma stem-cell samples; GDSC/pRRophetic drug-response prediction; U87/U251 *KIF20A*-knockdown models for proliferation/invasion/EMT.	*KIF20A* KD validated effects on proliferation, invasion and EMT, but *KIF20A* was not perturbed during TMZ treatment and no drug-response rescue experiment was performed.	Not tested by direct *KIF20A* perturbation under TMZ.	Retrospective/computational treatment-response association, including TMZ-sensitive/resistant expression data and survival analyses stratified by TMZ treatment; no prospective predictive validation.	D	[41]
*KIF3C*	Cargo transport/trafficking	Lung squamous cell carcinoma	Paclitaxel; cisplatin; gemcitabine	VASH2-induced multidrug resistance; an acquired paclitaxel-resistant line was also used for upstream VASH2 validation	VASH2-driven tubulin detyrosination increases *KIF3C* binding to microtubules and *KIF3C*-dependent EGFR endosomal recycling, sustaining PI3K/*Akt*/mTOR signaling.	H520 cells with VASH2 overexpression ± *KIF3C* knockdown; SK-MES-1 paclitaxel-resistant cells; LUSC cohorts; H520-VASH2 xenografts.	*KIF3C* KD + EGFR interaction/recycling assays + signaling analysis; no direct *KIF3C* inhibitor tested.	Yes, for VASH2-induced resistance in vitro; direct *KIF3C* reversal was not tested in the established SK-MES-1 paclitaxel-resistant line.	*KIF3C* KD reduced VASH2-driven xenograft growth/signaling; no *KIF3C*-specific in vivo chemotherapy-resistance reversal or clinical response validation.	B	[42]
*KIF14*	Mixed/multifunctional	Triple-negative breast cancer	Docetaxel	Baseline/intrinsic chemosensitivity and clinical chemoresistance association; no established acquired resistant cell line	*KIF14* supports docetaxel resistance through *AKT* activation. *KIF14* depletion lowers *AKT* phosphorylation, while re-expression of *KIF14* or constitutively active *AKT* rescues the chemosensitized phenotype, supporting an *AKT*-dependent mechanism.	TNBC cell lines with *KIF14* KD/OE; noncancerous HME1 cells with ectopic *KIF14*; a locally advanced TNBC neoadjuvant cohort (68 cases, including pCR and chemotherapy-resistant disease).	KD + OE + *KIF14* re-expression rescue + constitutively active *AKT* rescue + experimental *KIF14* ATPase inhibitor (*KIF14*i) + *AKT* localization/signaling assays.	Yes in non-established-resistance models: *KIF14* KD or *KIF14*i sensitized TNBC cells to docetaxel, whereas *KIF14* OE increased docetaxel resistance; *KIF14* or active *AKT* re-expression rescued sensitization. No reversal of a pre-established acquired resistant line was tested.	Clinical association: higher *KIF14* mRNA correlated with chemotherapy-resistant TNBC and lower expression with pCR. No in vivo chemotherapy-resistance reversal or prospective predictive validation.	B	[43]
*KIF15*	Mixed/multifunctional	Castration-resistant prostate cancer	Enzalutamide	Acquired enzalutamide resistance	*KIF15* directly binds the N-terminus of AR/AR-V7 and promotes USP14 association, preventing AR/AR-V7 degradation and sustaining AR signaling. Transcriptionally active AR reciprocally induces *KIF15* expression, forming a positive-feedback loop.	Enzalutamide-resistant prostate-cancer cells and resistant xenografts; AR/AR-V7 protein-interaction and stability models.	Genetic *KIF15* perturbation + pharmacological *KIF15* inhibition + AR/AR-V7 binding, USP14-association and protein-stability assays.	Yes. *KIF15* inhibition alone or combined with enzalutamide suppressed growth of enzalutamide-resistant prostate-cancer cells.	Yes. *KIF15* inhibition alone or in combination with enzalutamide suppressed progression of enzalutamide-resistant xenografts; no treatment-response patient cohort was validated in this study.	A	[44]
*KIFC2*	Non-canonical signaling/protein regulation	HR+/HER2− breast cancer	Tamoxifen; abemaciclib; palbociclib	Baseline sensitivity modulation plus acquired tamoxifen/palbociclib resistance	*KIFC2* recruits *USP9X* to deubiquitinate and stabilize *CDK4*, sustaining RB/*E2F*-associated cell-cycle output and resistance to endocrine therapy/*CDK4*/6 inhibition.	MCF7 and T47D KD/OE models; MCF7-TamR and MCF7-PalboR cells; xenografts; patient-derived organoids; FUSCC/TCGA/METABRIC cohorts.	KD + OE + protein-interaction/ubiquitination assays + *CDK4* re-expression rescue; pharmacological *USP9X* inhibition.	Yes; *KIFC2* depletion sensitized cells, while *CDK4* re-expression partially restored resistance; *USP9X* inhibition resensitized resistant models.	*KIFC2* KD improved tamoxifen/abemaciclib response in xenografts; PDO support and treatment-associated survival data, although the Palbo subgroup was small.	A	[45]
*KIF18B*	Non-canonical signaling/protein regulation	Colorectal cancer	Oxaliplatin	Acquired/established oxaliplatin-resistant phenotype	*KIF18B* interacts with *SP1* and disrupts *SP1*-mediated recruitment of *DNMT3B* to the *PARPBP* promoter, reducing promoter methylation and increasing *PARPBP* transcription; elevated *PARPBP* sustains oxaliplatin resistance in resistant CRC cells.	Oxaliplatin-resistant CRC cells versus non-resistant controls; CRC tissue analyses.	Functional manipulation of the *KIF18B*-*PARPBP* axis + *KIF18B*-*SP1* interaction + *DNMT3B* recruitment and *PARPBP* promoter methylation/transcriptional analyses. Exact perturbation modalities are not fully specified in the available abstract.	Supported at the cellular level. The study links *KIF18B*-induced *PARPBP* expression to maintenance of the resistant phenotype, although the exact resistance-reversal design is not fully detailed in the available abstract.	Clinical analyses showed a positive *KIF18B*-*PARPBP* relationship and poor prognosis with high *KIF18B* or *PARPBP*; no in vivo oxaliplatin-resistance reversal is reported in the available abstract.	B	[46]
*KIF14*/*KIF23*/*KIF18B*/*KIF24*/*KIFC1*	Mitotic/microtubule-associated KIF signature	Gastric cancer	Docetaxel; cabazitaxel	Predominantly intrinsic taxane resistance	Taxane-resistant diffuse-type gastric cancer shows impaired drug–microtubule engagement and coordinated modulation of five kinesins associated with taxane sensitivity; causality of individual KIFs was not established.	Post hoc TAX-325 clinical analysis; taxane-sensitive and taxane-resistant gastric cancer cell lines; paired baseline/on-treatment gastric cancer biopsies.	Transcriptomic/expression association only for the individual KIFs; no KIF-specific KD/OE/pharmacological perturbation.	Not tested for individual kinesins.	Clinical taxane-response association at the level of tumor subtype/drug–target engagement and KIF expression; no individual KIF-specific predictive validation.	D	[47]
*CENPE*/*KIF16B*/*KIF21A*	Mixed downstream kinesin/motor program	Cervical cancer	Paclitaxel; vincristine	Upstream *NSUN2*/*YBX1*–m5C–*SERPINB5*-mediated tolerance; individual kinesin-mediated resistance not established	*NSUN2*/*YBX1*-dependent m5C stabilization of *SERPINB5* induces a mitosis-centric program including *CENPE*, *KIF16B* and *KIF21A*, together with *CDK1*/CCNB1, thereby opposing microtubule-targeting drug-induced mitotic arrest; individual kinesin causality was not tested.	HeLa and SiHa models with *SERPINB5* knockdown, *NSUN2* knockout and *SERPINB5* re-expression rescue; scRNA-seq/spatial datasets; xenograft tumor-growth model; retrospectively classified paclitaxel-resistant versus sensitive cervical tumor specimens.	Strong functional evidence for the *NSUN2*–*SERPINB5* axis (KD/KO + rescue + drug IC50 assays). For *CENPE*/*KIF16B*/*KIF21A*, evidence was limited to downstream expression/PPI/correlation analyses and *CENPE* IHC; no KIF-specific KD/OE or pharmacological resistance test.	Yes for the upstream *NSUN2*–*SERPINB5* axis; not demonstrated for any individual kinesin.	Xenografts support *SERPINB5* oncogenicity and show reduced *CENPE* after *SERPINB5* depletion; patient tumors showed higher *SERPINB5* in paclitaxel-resistant cases. No individual kinesin-specific in vivo or clinical resistance validation.	D	[48]
*KIF23*	Mixed/multifunctional	HPV-negative head and neck squamous cell carcinoma	Cisplatin	Acquired cisplatin resistance (SCC9/CR and SCC25/CR; ~4-fold resistance) plus *SHCBP1*-driven resistance	*SHCBP1* physically interacts with *KIF23* and depends on *KIF23* for efficient nuclear localization. The *SHCBP1*-*KIF23* axis sustains cell-cycle progression and nuclear NF-kB/p65 and Wnt/beta-catenin signaling; *KIF23* depletion impairs *SHCBP1*-induced cisplatin resistance. *KIF23* depletion did not abolish *SHCBP1*-induced *Akt*/ERK activation.	Parental SCC9/SCC25 cells and acquired cisplatin-resistant SCC9/CR and SCC25/CR derivatives generated by ~6 months of stepwise/pulsed cisplatin exposure; *SHCBP1* KD/OE and *KIF23* KD models; SCC9/CR xenografts for the upstream *SHCBP1*-cisplatin axis.	*KIF23* KD + *SHCBP1* KD/OE + co-IP/localization studies + TCF/LEF reporter and signaling assays. *KIF23* KD partly abrogated *SHCBP1*-enhanced proliferation/cell-cycle progression and *SHCBP1*-induced cisplatin resistance; combined *SHCBP1*/*KIF23* depletion showed additive effects in several assays.	Partial/conditional. *KIF23* KD abrogated *SHCBP1*-induced cisplatin resistance in vitro, but direct resensitization of established SCC9/CR or SCC25/CR cells by *KIF23* depletion alone was not demonstrated.	The study used cisplatin-resistant SCC9/CR xenografts to validate *SHCBP1*-dependent cisplatin response, but *KIF23*-specific resistance reversal was not tested in vivo; no clinical treatment-response cohort for *KIF23*.	B	[49]
*KIF14*	Mixed/multifunctional	Hepatocellular carcinoma	Sorafenib	Acquired	Chronic sorafenib exposure activates an *AKT*-*ETS1*-*KIF14* positive-feedback loop. *KIF14* sustains *AKT* activation and *ETS1* expression, thereby maintaining the acquired resistant state.	Huh7-SR and HepG2-SR cells generated by chronic sorafenib exposure versus parental counterparts; sorafenib-resistant HCC xenografts.	Three *KIF14* siRNAs + sorafenib dose–response and apoptosis assays + *AKT*/*ETS1* pathway perturbation. A coefficient of drug interaction (CDI) analysis showed synergism between siKIF14 and sorafenib in resistant cells (best CDI ~0.66–0.69), but not in parental cells.	Yes. *KIF14* silencing resensitized established resistant cells to sorafenib and produced formally quantified synergistic effects with sorafenib in the resistant models.	Yes; siKIF14 combined with sorafenib suppressed growth of sorafenib-resistant HCC tumors in mice. No direct clinical validation; *KIF14* re-expression rescue was not performed.	A	[50]
*KIF18A*	Mitotic/microtubule-regulatory	Cholangiocarcinoma	Gemcitabine	Established acquired gemcitabine-resistant derivative (KKU-213B^GemR); direct *KIF18A*-mediated gemcitabine resistance was not supported	*KIF18A* sustains proliferation, migration, invasion and survival of gemcitabine-resistant CCA cells, with PI3K/*Akt*/mTOR, NF-κB and Bcl-2 signaling reduced after *KIF18A* depletion; however, these effects reflect survival/progression rather than a demonstrated gemcitabine-resistance mechanism.	Parental KKU-213B and gemcitabine-resistant KKU-213B^GemR cells; CRISPR/Cas9 *KIF18A* knockdown; gemcitabine MTT assays at 48/72 h; CCA tissue microarray (n = 84).	CRISPR/Cas9 KD + colony formation, migration/invasion, cell-cycle and Annexin V/PI apoptosis assays + pathway immunoblotting + direct gemcitabine dose–response testing; no OE or rescue experiment.	No; *KIF18A* knockdown did not significantly increase gemcitabine sensitivity in KKU-213B^GemR cells.	No in vivo validation. Patient tissues showed higher *KIF18A* in CCA than adjacent tissue and associations with tumor size/histology, but no OS association and no treatment-response validation.	C	[51]
KIFC3/*KIF5A*/*KIF12*	Mixed motor/microtubule-regulatory	Basal-like breast cancer	Docetaxel; paclitaxel	Clinical/intrinsic taxane-resistance association plus experimentally induced gain-of-function resistance	Overexpression of KIFC3, *KIF5A* or *KIF12* specifically increases taxane resistance. Resistance requires kinesin ATPase/motor activity: an ATP-binding-defective *KIF5A* mutant loses the ability to confer docetaxel resistance, supporting an ATPase-dependent motor mechanism that counteracts taxane-induced microtubule stabilization.	NCI-60 drug-response/expression data; pretreatment BLBC cohorts receiving taxane-containing or non-taxane regimens; residual tumors after neoadjuvant taxane therapy; MDA-MB-231/MDA-MB-468 cells stably overexpressing KIFC3, *KIF5A* or *KIF12*; *KIF5A* ATP-binding mutant.	Gain-of-function OE demonstrating sufficiency + drug-specificity testing + ATP-binding-domain mutant. No kinesin KD, pharmacological target inhibition or rescue of a pre-established resistant line.	No direct reversal of an established resistant phenotype. However, loss of ATPase function abolished *KIF5A*-mediated docetaxel resistance, demonstrating mechanistic necessity for the motor domain in the induced-resistance model.	Human cohort associations: KIFC3/*KIF5A* expression predicted poor paclitaxel response in taxane-treated BLBC cohorts, and *KIF12* was enriched in residual taxane-resistant tumors. No prospective validation or in vivo resistance-reversal study.	B	[52]
*KIF18A*	Mitotic/microtubule-regulatory	Early ER-positive breast cancer	Adjuvant endocrine therapy	Clinical poor-benefit/putative endocrine resistance; causality not established	No direct endocrine-resistance mechanism was experimentally demonstrated. High *KIF18A* mRNA/protein was associated with poorer outcomes in endocrine-treated patients, but similar adverse associations were also present in untreated patients, indicating substantial prognostic rather than treatment-specific information.	METABRIC ER-positive cohort (n = 1506); KM-Plot validation (n = 2061); large early-stage ER-positive breast-cancer IHC cohort (n = 1592). No endocrine-resistant cell model was used.	Observational genomic/transcript/protein expression and multivariable survival analyses only; no *KIF18A* KD/OE, endocrine-drug sensitivity assay, rescue experiment or pharmacological inhibition.	Not tested.	Clinical association only. High *KIF18A* was associated with poor RFS, DMFS and breast-cancer-specific survival in endocrine-treated patients, but also with adverse outcomes in untreated patients; no treatment-by-biomarker interaction or prospective predictive validation was reported.	D	[53]

Evidence levels: A, strong causal evidence supported by established resistant models, functional perturbation/resensitization, mechanistic validation, and/or in vivo validation; B, functional evidence that kinesin perturbation changes drug response but lacks one or more major causal/translational elements; C, limited or discordant functional evidence, including sensitization without a pre-established resistant model or studies in resistant cells in which direct resistance reversal is absent or not supported; D, associative or indirect evidence without direct functional demonstration that the individual kinesin mediates resistance. KD, knockdown; OE, overexpression; MTA, microtubule-targeting agent; DDP, cisplatin; PDO, patient-derived organoid. Reference numbers follow the manuscript bibliography.

**Table 2:** Epigenetic, Transcriptional, and RNA-Based Regulation Associated with Kinesin-Related Therapeutic Resistance.

Regulatory Layer	Upstream Regulator	Kinesin-Related Target	Cancer Type	Therapy/Resistance Context	Proposed Mechanism	Evidence Interpretation	Ref.
Transcriptional regulation	*JNK1*/*c-Jun*	*KIF18A*	Cervical cancer	Not therapy-specific	*c-Jun* directly activates *KIF18A* transcription	Regulation established; resistance dependency not tested	[58]
Chromatin-associated transcription	*ANCCA*/ATAD2	*KIF4A*/*KIF15*/*KIF20A*/*KIF23*	ER+ breast cancer	Relapse/endocrine-associated context	Estrogen-dependent coordinated kinesin transcription	Clinical association; individual resistance dependencies unvalidated	[59]
Histone modification/chromatin remodeling	*NSD2*	*KIF18A*	mCRPC	Castration-resistant disease evolution	H3K36me2 gain/H3K27me3 loss and compartment activation induce *KIF18A*	Epigenetic activation during resistant evolution; no direct *KIF18A*-dependent ADT reversal	[60]
Transcriptional/redox circuit	*YY1*	*KIF15*–*PRDX1*	Gastric cancer	Cisplatin resistance	*YY1* activates *KIF15*; *KIF15* stabilizes *PRDX1* and maintains mitochondrial redox homeostasis	Functional resistance-associated circuit with rescue/in vivo support	[61]
DNA methylation	*SP1*/*DNMT3B*	*KIF18B*–*PARPBP*	CRC	Oxaliplatin resistance	*KIF18B* reduces *DNMT3B* recruitment and *PARPBP* promoter methylation	Functional epigenetic resistance pathway	[46]
m6A modification	*METTL3*	*KIF9-AS1* (lncRNA)	HCC	Sorafenib resistance	m6A stabilizes *KIF9-AS1* → U*SP1*/*SHOX2* → stemness	Direct epitranscriptomic kinesin-associated lncRNA pathway; *KIF9-AS1* is not a motor protein	[62]
m5C regulation	Hypoxia/*ALYREF*	*KIF20A*–*BUB1*	Cervical cancer	Ferroptosis resistance	m5C-dependent activation of *KIF20A*/*BUB1* suppresses ferroptotic vulnerability	Functional RNA-modification-associated *KIF20A* pathway	[65]
RNA modification	*NSUN2*/*YBX1*	*SERPINB5* → *CENPE*/*KIF16B*/*KIF21A*	Cervical cancer	Paclitaxel/vincristine resistance	m5C stabilizes *SERPINB5* and induces motor-associated program	Indirect kinesin-associated evidence; individual KIFs not tested	[48]
lncRNA/miRNA regulation	*AC105118.1*/*miR-378a-3p*	*KIF26B*	CRC	Oxaliplatin resistance	lncRNA-mediated miRNA sequestration releases *KIF26B* and increases glycolysis	Direct ncRNA–KIF resistance axis	[63]
miRNA regulation	*miR-127-3p*	*KIF3B*	TNBC	Docetaxel resistance	*miR-127-3p* loss permits *KIF3B*-dependent stemness	Direct miRNA–KIF resistance axis with *KIF3B* rescue	[64]

## Data Availability

No new data were created or analyzed in this study. Data sharing is not applicable to this article.

## Supplementary Materials

The following supporting information can be downloaded at: https://www.mdpi.com/article/10.3390/cancers18172872/s1. Supplementary Table S1. Expression patterns and clinical associations of kinesin family members across human cancers [148,149,150,151,152,153,154,155,156,157,158,159,160,161,162,163,164,165,166,167,168,169,170,171,172,173,174,175,176,177,178,179,180,181,182,183,184,185,186,187,188,189,190,191,192,193,194,195].

## Abbreviations

The following abbreviations are used in this manuscript:

3D-QSAR	Three-dimensional quantitative structure–activity relationship
AJCC	American Joint Committee on Cancer
AI	Artificial intelligence
AR	Androgen receptor
AR-V7	Androgen receptor splice variant 7
ATP	Adenosine triphosphate
BCSS	Breast cancer-specific survival
CCC	Ovarian clear cell carcinoma
CDX	Cell line-derived xenograft
CENP-E	Centromere protein E
CNV	Copy number variation
CRISPR/Cas9	Clustered regularly interspaced short palindromic repeats/CRISPR-associated protein 9
cryo-EM	Cryo-electron microscopy
CSC	Cancer stem cell
DDR	DNA damage response
DFS	Disease-free survival
DHA	Dihydroartemisinin
DHPM	Dihydropyrimidinone
DLBCL	Diffuse large B-cell lymphoma
DMFS	Distant metastasis-free survival
EMT	Epithelial–mesenchymal transition
FIGO	International Federation of Gynecology and Obstetrics
HCC	Hepatocellular carcinoma
HIF	Hypoxia-inducible factor
iPSC	Induced pluripotent stem cell
KIF	Kinesin family member
LN	Lymph node
LSTM	Long short-term memory
LUAD	Lung adenocarcinoma
NPC	Nasopharyngeal carcinoma
MCAK	Mitotic centromere-associated kinesin
MM-GBSA	Molecular mechanics/generalized Born surface area
NSCLC	Non-small cell lung cancer
OS	Overall survival
PDX	Patient-derived xenograft
PFS	Progression-free survival
PPI	Protein–protein interaction
PSA	Prostate-specific antigen
RCC	Renal cell carcinoma
RFS	Relapse-free survival
RNA-seq	RNA sequencing
SAC	Spindle assembly checkpoint
SAR	Structure–activity relationship
TCGA	The Cancer Genome Atlas
TMZ	The Cancer Genome Atlas
TNBC	Triple-negative breast cancer

## References

[1] Zaniewski T.M., Hancock W.O. (2023). Positive charge in the K-loop of the kinesin-3 motor *KIF1A* regulates superprocessivity by enhancing microtubule affinity in the one-head-bound state. J. Biol. Chem..

[2] Kumar R., Madhavan T., Ponnusamy K., Sohn H., Haider S. (2023). Computational study of the motor neuron protein KIF5A to identify nsSNPs, bioactive compounds, and its key regulators. Front. Genet..

[3] Hassan Ibrahim I., Balah A., Gomaa Abd Elfattah Hassan A., Gamal Abd El-Aziz H. (2022). Role of motor proteins in human cancers. Saudi J. Biol. Sci..

[4] Rieu M., Krutyholowa R., Taylor N.M.I., Berry R.M. (2022). A new class of biological ion-driven rotary molecular motors with 5:2 symmetry. Front. Microbiol..

[5] Cushion T.D., Leca I., Keays D.A. (2023). MAPping tubulin mutations. Front. Cell Dev. Biol..

[6] Loncar A., Rincon S.A., Lera Ramirez M., Paoletti A., Tran P.T. (2020). Kinesin-14 family proteins and microtubule dynamics define *S. pombe* mitotic and meiotic spindle assembly, and elongation. J. Cell Sci..

[7] Malaby H.L.H., Dumas M.E., Ohi R., Stumpff J. (2019). Kinesin-binding protein ensures accurate chromosome segregation by buffering KIF18A and KIF15. J. Cell Biol..

[8] Kalantari S., Carlston C., Alsaleh N., Abdel-Salam G.M.H., Alkuraya F., Kato M., Matsumoto N., Miyatake S., Yamamoto T., Fares-Taie L. (2021). Expanding the *KIF4A*-associated phenotype. Am. J. Med. Genet. Part A.

[9] Koonce M.P., Tikhonenko I. (2020). Bioenergetics of the *Dictyostelium* Kinesin-8 Motor Isoform. Biomolecules.

[10] Zhao J., Fok A.H.K., Fan R., Kwan P., Chan H., Lo L.H., Chan Y., Yung W., Huang J., Lai C.S.W. (2020). Specific depletion of the motor protein KIF5B leads to deficits in dendritic transport, synaptic plasticity and memory. eLife.

[11] Soppina P., Shewale D.J., Naik P.K., Soppina V. (2022). Single-Molecule Analysis of Sf9 Purified Superprocessive Kinesin-3 Family Motors. J. Vis. Exp..

[12] Hou J., Shao X., Zhang Y., Jin F., Xu W., Xu X. (2026). Oncogenic Role of *KIF18B* Across Human Cancers: A Pan-Cancer Bioinformatic Analysis and Experimental Validation in Lung Adenocarcinoma. Front. Biosci. (Landmark Ed.).

[13] Wu H., Duan Y., Gong S., Zhu Q., Liu X., Liu Z. (2022). An Integrative Pan-Cancer Analysis of Kinesin Family Member C1 (*KIFC1*) in Human Tumors. Biomedicines.

[14] Wang Q., Zhou Y., Zhou Ge Y., Qin G., Yin T., Zhao D., Tan C., Yao S. (2022). Comprehensive proteomic signature and identification of *CDKN2A* as a promising prognostic biomarker and therapeutic target of colorectal cancer. World J. Clin. Cases.

[15] Guo X., Zhou L., Wu Y., Li J. (2022). *KIF11* as a potential pan-cancer immunological biomarker encompassing the disease staging, prognoses, tumor microenvironment, and therapeutic responses. Oxid. Med. Cell. Longev..

[16] Wang H., Zhou L., Huang F. (2025). Kinesin genes *KIF4A*, *KIF20A* and *KIF11* as prognostic biomarkers in lung adenocarcinoma by integrative bioinformatic analysis and experimental validation. Sci. Rep..

[17] Marescal O., Cheeseman I.M. (2025). 19S proteasome loss regulates mitotic spindle assembly through a ubiquitin-independent degradation mechanism. Cell Rep..

[18] Su Z., Zhong Y., He Y., You L., Xin F., Wang L., Liu Z. (2024). Bulk- and single cell-RNA sequencing reveal *KIF20A* as a key driver of hepatocellular carcinoma progression and immune evasion. Front. Immunol..

[19] Lucanus A.J., Yip G.W. (2018). Kinesin superfamily: Roles in breast cancer, patient prognosis and therapeutics. Oncogene.

[20] Bishoyi A.K., Al-Hasnaawei S., Ganesan S., Shankhyan A., Nanda A., Sinha A., Ray S., Nathiya D. (2026). *KIF14* in cancer biology: Implications for diagnosis and therapy. Clin. Exp. Metastasis.

[21] Wang J., Cui F., Wang X., Xue Y., Chen J., Yu Y., Lu H., Zhang M., Tang H., Peng Z. (2015). Elevated kinesin family member 26B is a prognostic biomarker and a potential therapeutic target for colorectal cancer. J. Exp. Clin. Cancer Res..

[22] Mbugua R.W., Takano A., Tsevegjav B., Yokose T., Yamashita T., Miyagi Y., Daigo Y. (2024). Characterization of *KIF20B* as a novel prognostic biomarker and therapeutic target for breast cancer. Int. J. Oncol..

[23] Fu X., Zhu Y., Zheng B., Zou Y., Wang C., Wu P., Wang J., Chen H., Du P., Liang B. (2018). *KIFC1*, a novel potential prognostic factor and therapeutic target in hepatocellular carcinoma. Int. J. Oncol..

[24] Kostecka L.G., Olseen A., Kang K., Torga G., Pienta K.J., Amend S.R. (2021). High *KIFC1* expression is associated with poor prognosis in prostate cancer. Med. Oncol..

[25] Nakano Y., Sekino Y., Kobayashi G., Nakahara H., Akabane S., Takemoto K., Naito M., Miyamoto S., Kobatake K., Kitano H. (2026). *KIFC1* is associated with sarcomatoid differentiation, immune response, and a poor prognosis in clear cell renal cell carcinoma. Cancer Med..

[26] Sekino Y., Pham Q.T., Kobatake K., Kitano H., Ikeda K., Goto K., Hayashi T., Nakahara H., Sentani K., Oue N. (2021). *KIFC1* Is Associated with Basal Type, Cisplatin Resistance, PD-L1 Expression and Poor Prognosis in Bladder Cancer. J. Clin. Med..

[27] Li X., Wang S., Ruan P., Bajinka O., Zhang W. (2024). High expression of *KIFC1* is a poor prognostic biomarker and correlates with *TP53* mutation in lung cancer. Medicine.

[28] Xue P., Zheng J., Li R., Yan L., Wang Z., Jia Q., Zhang L., Li X. (2024). High expression of *KIFC1* in glioma correlates with poor prognosis. J. Korean Neurosurg. Soc..

[29] Li T., Zhai D., Zhang M., Ye R., Kuang X., Shao N., Bi J., Lin Y. (2022). *KIF17* maintains the epithelial phenotype of breast cancer cells and curbs tumour metastasis. Cancer Lett..

[30] Yang Y., Liu X., Li R., Zhang M., Wang H., Qu Y. (2020). Kinesin family member 3A inhibits the carcinogenesis of non-small cell lung cancer and prolongs survival. Oncol. Lett..

[31] Wang F., Fu X., Chang M., Wei T., Lin R., Tong H., Zhang X., Yuan R., Zhou Z., Huang X. (2024). The interaction of calcium-sensing receptor with *KIF11* enhances cisplatin resistance in lung adenocarcinoma via *BRCA1*/cyclin B1 pathway. Int. J. Biol. Sci..

[32] Pao Y., Liao K., Shiau Y., Chao M., Li M., Lin L., Chang H., Yeh H., Chen Y., Chiu Y. (2025). *KIF2C* promotes paclitaxel resistance by depolymerizing polyglutamylated microtubules. Dev. Cell.

[33] Ganguly A., Yang H., Cabral F. (2011). Overexpression of mitotic centromere-associated kinesin stimulates microtubule detachment and confers resistance to paclitaxel. Mol. Cancer Ther..

[34] Liu L., Li M., Zhang J., Xu D., Guo Y., Zhang H., Cang S. (2023). *KIF14* mediates cabazitaxel-docetaxel cross-resistance in advanced prostate cancer by promoting AKT phosphorylation. Arch. Biochem. Biophys..

[35] Su Y., Liu X., Yu Y., Chen X., Shi L., Du Z., Mao Y., Yin F. (2026). Low *KIF26B* expression reduces paclitaxel resistance and predicts good prognosis in ovarian cancer. Curr. Issues Mol. Biol..

[36] Huang Y., Yuan C., Liu Q., Wang L. (2022). *KIF23* promotes autophagy-induced imatinib resistance in chronic myeloid leukaemia through activating Wnt/β-catenin pathway. Clin. Exp. Pharmacol. Physiol..

[37] Wan Q., Shen Y., Zhao H., Wang B., Zhao L., Zhang Y., Bu X., Wan M., Shen C. (2019). Impaired DNA double-strand breaks repair by kinesin family member 4A inhibition renders human H1299 non-small-cell lung cancer cells sensitive to cisplatin. J. Cell. Physiol..

[38] Zhou Y., Yang L., Xiong L., Wang K., Hou X., Li Q., Kong F., Liu X., He J. (2021). *KIF11* is upregulated in colorectal cancer and silencing of it impairs tumor growth and sensitizes colorectal cancer cells to oxaliplatin via p53/GSK3β signaling. J. Cancer.

[39] Liu B., Zhang G., Cui S., Du G. (2022). Upregulation of *KIF11* in *TP53* mutant glioma promotes tumor stemness and drug resistance. Cell. Mol. Neurobiol..

[40] Wang J., Cai C., Zhang X., Ge C., Zhou S., Jin Z., Dai K., Dai N., Yu X., Wang J. (2025). *KIF4A* facilitates oxaliplatin resistance and stemness in colon cancer by boosting glucose metabolism. Digestion.

[41] Ye L., Tong S., Wang Y., Wang Y., Ma W. (2023). Grade scoring system reveals distinct molecular subtypes and identifies *KIF20A* as a novel biomarker for predicting temozolomide treatment efficiency in gliomas. J. Cancer Res. Clin..

[42] Wang J., Liu P., Zhang R., Xing B., Chen G., Han L., Yu J. (2024). VASH2 enhances *KIF3C*-mediated EGFR-endosomal recycling to promote aggression and chemoresistance of lung squamous cell carcinoma by increasing tubulin detyrosination. Cell Death Dis..

[43] Singel S.M., Cornelius C., Zaganjor E., Batten K., Sarode V.R., Buckley D.L., Peng Y., John G.B., Li H.C., Sadeghi N. (2014). *KIF14* promotes AKT phosphorylation and contributes to chemoresistance in triple-negative breast cancer. Neoplasia.

[44] Gao L., Zhang W., Zhang J., Liu J., Sun F., Liu H., Hu J., Wang X., Wang X., Su P. (2021). *KIF15*-mediated stabilization of AR and AR-V7 contributes to enzalutamide resistance in prostate cancer. Cancer Res..

[45] Yang S., Jin M., Andriani L., Zhao Q., Ling Y., Lin C., Huang M., Cai J., Zhang Y., Hu X. (2025). Kinesin-like protein KIFC2 stabilizes CDK4 to accelerate growth and confer resistance in HR^+^/HER2^−^ breast cancer. J. Clin. Investig..

[46] Hong B., Lu R., Lou W., Bao Y., Qiao L., Hu Y., Liu K., Chen J., Bao D., Ye M. (2021). *KIF18b*-dependent hypomethylation of *PARPBP* gene promoter enhances oxaliplatin resistance in colorectal cancer. Exp. Cell Res..

[47] Galletti G., Zhang C., Gjyrezi A., Cleveland K., Zhang J., Powell S., Thakkar P.V., Betel D., Shah M.A., Giannakakou P. (2020). Microtubule engagement with taxane is altered in taxane-resistant gastric cancer. Clin. Cancer Res..

[48] Liu J., Zhou L., Yao P., Zhang N., Guo X., Chen F., Yang S., Du X., Wang H., Zhou Y. (2026). M^5^C-driven stabilization of *SERPINB5* promotes cervical cancer progression and chemotherapy resistance. Cell Death Dis..

[49] Sun Y., Pan H., He Y., Hu C., Gu Y. (2022). Functional roles of the *SHCBP1* and *KIF23* interaction in modulating the cell-cycle and cisplatin resistance of head and neck squamous cell carcinoma. Head Neck.

[50] Zhu Q., Ren H., Li X., Qian B., Fan S., Hu F., Xu L., Zhai B. (2020). Silencing *KIF14* reverses acquired resistance to sorafenib in hepatocellular carcinoma. Aging.

[51] Tanasuka P., Thongpon P., Chomwong S., Kongsintaweesuk S., Pinlaor S., Pongking T., Watcharadetwittaya S., Pairojkul C., Intuyod K. (2025). Suppression of kinesin family member-18A diminishes progression and induces apoptotic cell death of gemcitabine-resistant cholangiocarcinoma cells by modulating PI3K/Akt/mTOR and NF-κB pathways. PLoS ONE.

[52] Tan M.H., De S., Bebek G., Orloff M.S., Wesolowski R., Downs-Kelly E., Budd G.T., Stark G.R., Eng C. (2012). Specific kinesin expression profiles associated with taxane resistance in basal-like breast cancer. Breast Cancer Res. Treat..

[53] Alfarsi L.H., Elansari R., Toss M.S., Diez-Rodriguez M., Nolan C.C., Ellis I.O., Rakha E.A., Green A.R. (2019). Kinesin family member-18A (*KIF18A*) is a predictive biomarker of poor benefit from endocrine therapy in early ER+ breast cancer. Breast Cancer Res. Treat..

[54] Zou Y., Shan M., Wan X., Liu J., Zhang K., Ju J., Xing C., Sun S. (2022). Kinesin *KIF15* regulates tubulin acetylation and spindle assembly checkpoint in mouse oocyte meiosis. Cell. Mol. Life Sci..

[55] Schlisio S., Kenchappa R.S., Vredeveld L.C.W., George R.E., Stewart R., Greulich H., Shahriari K., Nguyen N.V., Pigny P., Dahia P.L. (2008). The kinesin KIF1Bbeta acts downstream from EglN3 to induce apoptosis and is a potential 1p36 tumor suppressor. Genes Dev..

[56] Wang D., Li M., Ma J., Du G., Zhou J., Chen J., Guan X. (2026). Mitocytosis, mitophagy, and apoptosis coordinately drive mitochondrial clearance to regulate myogenic differentiation. J. Adv. Res..

[57] Sun D., Li X., Nie S., Liu J., Wang S. (2023). Disorders of cancer metabolism: The therapeutic potential of cannabinoids. Biomed. Pharmacother..

[58] Wang Y., Zhou B., Lian X., Yu S., Huang B., Wu X., Wen L., Zhu C. (2025). *KIF18A* is a novel target of JNK1/c-Jun signaling pathway involved in cervical tumorigenesis. J. Cell. Physiol..

[59] Zou J.X., Duan Z., Wang J., Sokolov A., Xu J., Chen C.Z., Li J.J., Chen H. (2014). Kinesin family deregulation coordinated by bromodomain protein ANCCA and histone methyltransferase MLL for breast cancer cell growth, survival, and tamoxifen resistance. Mol. Cancer Res..

[60] Kanaoka S., Okabe A., Kanesaka M., Rahmutulla B., Fukuyo M., Seki M., Hoshii T., Sato H., Imamura Y., Sakamoto S. (2024). Chromatin activation with H3K36me2 and compartment shift in metastatic castration-resistant prostate cancer. Cancer Lett..

[61] Li W., Wu B., Qian H., Zhang G., Wang X., Li X. (2026). The *YY1-KIF15-PRDX1* axis promotes gastric cancer progression by inducing mitochondrial ROS imbalance. Oncogene.

[62] Yu Y., Lu X., Mu J., Meng J., Sun J., Chen H., Yan Y., Meng K. (2024). N6-methyladenosine-modified long non-coding RNA *KIF9-AS1* promotes stemness and sorafenib resistance in hepatocellular carcinoma by upregulating *SHOX2* expression. World J. Gastroenterol..

[63] Zhang Y., Shen Z., Han X., Wu Y., Huang T. (2024). Long non-coding RNA AC105118.1 affects glycolysis to facilitate oxaliplatin resistance in colorectal cancer cells by modulating the miR-378a-3p/*KIF26B* axis. Int. J. Biochem. Cell Biol..

[64] He Z., Zhao J., Yan T. (2026). miR-127-3p inhibits cell stemness and docetaxel resistance in triple-negative breast cancer by targeting *KIF3B*. Kaohsiung J. Med. Sci..

[65] Gao Y., Zou T. (2026). Hypoxia triggers *ALYREF*-mediated m^5^C methylation of *KIF20A* to activate *KIF20A/BUB1* for generating ferroptosis resistance in cervical cancer cells. Kaohsiung J. Med. Sci..

[66] Zheng J., She H., Han R., Tang J., Dou Y., Lu C., Huang D., Lin C., Wu D., He C. (2025). *Dapk2* dysfunction leads to Mic60 lactylation and mitochondrial metabolic reprogramming, promoting lung cancer EGFR-TKI resistance and metastasis. Dev. Cell.

[67] Pu J., Keren-Kaplan T., Bonifacino J.S. (2017). A Ragulator-BORC interaction controls lysosome positioning in response to amino acid availability. J. Cell Biol..

[68] Li S., Fell S.M., Surova O., Smedler E., Wallis K., Chen Z.X., Hellman U., Johnsen J.I., Martinsson T., Kenchappa R.S. (2016). The 1p36 tumor suppressor KIF1Bβ is required for calcineurin activation, controlling mitochondrial fission and apoptosis. Dev. Cell.

[69] Ando K., Yokochi T., Mukai A., Wei G., Li Y., Kramer S., Ozaki T., Maehara Y., Nakagawara A. (2019). Tumor suppressor KIF1Bβ regulates mitochondrial apoptosis in collaboration with *YME1L1*. Mol. Carcinog..

[70] Baek J., Lee J., Yun H.S., Lee C., Song J., Um H., Park J.K., Park I., Kim J., Kim E.H. (2018). Kinesin light chain-4 depletion induces apoptosis of radioresistant cancer cells by mitochondrial dysfunction via calcium ion influx. Cell Death Dis..

[71] Yang C., Zhang Y., Lin S., Liu Y., Li W. (2021). Suppressing the *KIF20A*/*NUAK1*/Nrf2/*GPX4* signaling pathway induces ferroptosis and enhances the sensitivity of colorectal cancer to oxaliplatin. Aging.

[72] Shen X., Cheng H., Zheng J., Xia Y., Li Y., Guo Q., Zhang Z., Yin N., Liu Y., Dong J. (2025). *KIF4A* inhibits ferroptosis in glioblastoma via the *CHMP4B/GPX4* axis and promotes temozolomide resistance. Mol. Carcinog..

[73] Zhang Z., Li Y., Hu J., Tang X., Li Z., Sun X., Feng D., Yao Y., Mao C., Tao Y. (2026). *KIF20A* inhibits *TRIM21*-dependent ubiquitination of *DHX9* to boost *SOX2* stability, enhancing OSCC stemness and ferroptosis resistance. Cell Death Dis..

[74] Hu H., Wang P., Kong Z., Yang Y., Liu S., Li G., Wang C., Jiang J., He Q., Cao X. (2026). *KIF20A* drives epithelial cell proliferation and migration in gastric adenocarcinoma, facilitating macrophage M2 polarization and subsequent immune evasion. Int. J. Biol. Macromol..

[75] Zhao X., Xuan F., Li Z., Yin X., Zeng X., Chen J., Fang C. (2025). A *KIF20A*-based thermosensitive hydrogel vaccine effectively potentiates immune checkpoint blockade therapy for hepatocellular carcinoma. npj Vaccines.

[76] Cho J.G., Hah Y., Yun E., Ka H.I., Lee A., Han S., Lee D., Kim S.W., Park J.H., Kwon B.S. (2025). Loss of primary cilia promotes EphA2-mediated endothelial-to-mesenchymal transition in the ovarian tumor microenvironment. Mol. Oncol..

[77] Yang M., Huang H., Zhang Y., Wang Y., Zhao J., Lee P., Ma Y., Qu S. (2024). Identification and validation of *KIF20A* for predicting prognosis and treatment outcomes in patients with breast cancer. Sci. Rep..

[78] Owida H.A., Mohammad S.I., Vasudevan A., Bishoyi A.K., RenukaJyothi S., Panigrahi R., Abosaoda M.K., Garg G., Pargaien A. (2025). Kinesin superfamily proteins in cancer: Unveiling their role in chemotherapy. Int. Immunopharmacol..

[79] Mayer T.U., Kapoor T.M., Haggarty S.J., King R.W., Schreiber S.L., Mitchison T.J. (1999). Small molecule inhibitor of mitotic spindle bipolarity identified in a phenotype-based screen. Science.

[80] Maliga Z., Kapoor T.M., Mitchison T.J. (2002). Evidence that monastrol is an allosteric inhibitor of the mitotic kinesin Eg5. Chem. Biol..

[81] Pena A., Sweeney A., Cook A.D., Locke J., Topf M., Moores C.A. (2020). Structure of microtubule-trapped human kinesin-5 and its mechanism of inhibition revealed using cryoelectron microscopy. Structure.

[82] Pfeffer T.J., Sasse F., Schmidt C.F., Lakämper S., Kirschning A., Scholz T. (2016). The natural diterpene tonantzitlolone A and its synthetic enantiomer inhibit cell proliferation and kinesin-5 function. Eur. J. Med. Chem..

[83] Blackburn C.L., Hopmann C., Sakowicz R., Berdelis M.S., Goldstein L.S.B., Faulkner D.J. (1999). Adociasulfates 1–6, inhibitors of kinesin motor proteins from the sponge *Haliclona* (aka *Adocia*) sp.. J. Org. Chem..

[84] Brier S., Carletti E., DeBonis S., Hewat E., Lemaire D., Kozielski F. (2006). The marine natural product adociasulfate-2 as a tool to identify the MT-binding region of kinesins. Biochemistry.

[85] Lee C.W., Bélanger K., Rao S.C., Petrella T.M., Tozer R.G., Wood L., Savage K.J., Eisenhauer E.A., Synold T.W., Wainman N. (2008). A phase II study of ispinesib (SB-715992) in patients with metastatic or recurrent malignant melanoma: A National Cancer Institute of Canada Clinical Trials Group trial. Investig. New Drugs.

[86] Beer T.M., Goldman B., Synold T.W., Ryan C.W., Vasist L.S., Van Veldhuizen P.J.J., Dakhil S.R., Lara P.N.J., Drelichman A., Hussain M.H.A. (2008). Southwest Oncology Group phase II study of ispinesib in androgen-independent prostate cancer previously treated with taxanes. Clin. Genitourin. Cancer.

[87] Knox J.J., Gill S., Synold T.W., Biagi J.J., Major P., Feld R., Cripps C., Wainman N., Eisenhauer E., Seymour L. (2008). A phase II and pharmacokinetic study of SB-715992, in patients with metastatic hepatocellular carcinoma: A study of the National Cancer Institute of Canada Clinical Trials Group (NCIC CTG IND.168). Investig. New Drugs.

[88] Kathman S.J., Williams D.H., Hodge J.P., Dar M. (2007). A Bayesian population PK-PD model of ispinesib-induced myelosuppression. Clin. Pharmacol. Ther..

[89] Reinemann D.N., Sturgill E.G., Das D.K., Degen M.S., Vörös Z., Hwang W., Ohi R., Lang M.J. (2017). Collective force regulation in anti-parallel microtubule gliding by dimeric KIF15 kinesin motors. Curr. Biol..

[90] Tanenbaum M.E., Macůrek L., Janssen A., Geers E.F., Alvarez-Fernández M., Medema R.H. (2009). KIF15 cooperates with Eg5 to promote bipolar spindle assembly. Curr. Biol..

[91] Ma H.T., Erdal S., Huang S., Poon R.Y.C. (2014). Synergism between inhibitors of Aurora A and KIF11 overcomes KIF15-dependent drug resistance. Mol. Oncol..

[92] Payton M., Belmontes B., Hanestad K., Moriguchi J., Chen K., McCarter J.D., Chung G., Ninniri M.S., Sun J., Manoukian R. (2024). Small-molecule inhibition of kinesin KIF18A reveals a mitotic vulnerability enriched in chromosomally unstable cancers. Nat. Cancer.

[93] Phillips A.F., Zhang R., Jaffe M., Schulz R., Carty M.C., Verma A., Feinberg T.Y., Arensman M.D., Chiu A., Letso R. (2025). Targeting chromosomally unstable tumors with a selective KIF18A inhibitor. Nat. Commun..

[94] Watts C.A., Richards F.M., Bender A., Bond P.J., Korb O., Kern O., Riddick M., Owen P., Myers R.M., Raff J. (2013). Design, synthesis, and biological evaluation of an allosteric inhibitor of HSET that targets cancer cells with supernumerary centrosomes. Chem. Biol..

[95] Sekino Y., Oue N., Koike Y., Shigematsu Y., Sakamoto N., Sentani K., Teishima J., Shiota M., Matsubara A., Yasui W. (2019). KIFC1 inhibitor CW069 induces apoptosis and reverses resistance to docetaxel in prostate cancer. J. Clin. Med..

[96] Kawaguchi K. (2013). Role of kinesin-1 in the pathogenesis of SPG10, a rare form of hereditary spastic paraplegia. Neuroscientist.

[97] Cai Q., Pan P., Sheng Z. (2007). Syntabulin-kinesin-1 family member 5B-mediated axonal transport contributes to activity-dependent presynaptic assembly. J. Neurosci..

[98] Milic B., Chakraborty A., Han K., Bassik M.C., Block S.M. (2018). KIF15 nanomechanics and kinesin inhibitors, with implications for cancer chemotherapeutics. Proc. Natl. Acad. Sci. USA.

[99] Lombard A.P., Liu C., Armstrong C.M., Cucchiara V., Gu X., Lou W., Evans C.P., Gao A.C. (2017). ABCB1 mediates cabazitaxel-docetaxel cross-resistance in advanced prostate cancer. Mol. Cancer Ther..

[100] Terribas E., Fernández M., Mazuelas H., Fernández-Rodríguez J., Biayna J., Blanco I., Bernal G., Ramos-Oliver I., Thomas C., Guha R. (2020). KIF11 and KIF15 mitotic kinesins are potential therapeutic vulnerabilities for malignant peripheral nerve sheath tumors. Neuro Oncol. Adv..

[101] Alam A.K.M.M., Cai X., Ullah Khan M.I., Musiol R., Shubhra Q.T.H. (2026). Mimic, target, and adapt: Cell membrane-coated nanoplatforms for precision and immune-guided cancer treatment. BMEMat.

[102] Shubhra Q.T.H., Musiol R., Pan H., Cai Q., He X., Cai X. (2025). Illuminating the unseen and targeting the untreatable: Aggregation-induced emission nanoparticles as intelligent, immune-compatible tools for precision cancer theranostics. Aggregate.

[103] Zhou Q., Shubhra Q.T.H., Lai P., Shi J., Fang C., Guo Q., Li W., Chen R., Shen X., Huang L. (2025). Genistein and chlorin E6-loaded versatile nanoformulation for remodeling the hypoxia-related tumor microenvironment and boosting photodynamic therapy in nasopharyngeal carcinoma treatment. Adv. Compos. Hybrid. Mater..

[104] Rios E.A.M., Dea C.M., Dos Santos E.R.F.B., D’Oca M.G.M., Rampon D.S., Nachtigall F.M., Santos L.S., Guzman L., Moore-Carrasco R., Rebolledo-Mira D. (2024). Synthesis of novel fatty acid 3,4-dihydropyrimidin-2-(1H)-one and antitumoral activity against breast and gastric cancer cells. RSC Adv..

[105] Jiang X., Chen S., Liu Y., Pan X., Chen D., Wang S. (2021). Solvothermal synthesis of multiple dihydropyrimidinones at a time as inhibitors of Eg5. Molecules.

[106] Liu J., Mallick S., Xie Y., Grassin C., Lucas B., Schölermann B., Pahl A., Scheel R., Strohmann C., Protzel C. (2023). Morphological profiling identifies the motor protein Eg5 as cellular target of spirooxindoles. Angew. Chem. Int. Ed..

[107] Pernigo S., Chegkazi M.S., Yip Y.Y., Treacy C., Glorani G., Hansen K., Politis A., Bui S., Dodding M.P., Steiner R.A. (2018). Structural basis for isoform-specific kinesin-1 recognition of Y-acidic cargo adaptors. eLife.

[108] Benoit M.P.M.H., Asenjo A.B., Sosa H. (2018). Cryo-EM reveals the structural basis of microtubule depolymerization by kinesin-13s. Nat. Commun..

[109] von Loeffelholz O., Moores C.A. (2019). Cryo-EM structure of the *Ustilago maydis* kinesin-5 motor domain bound to microtubules. J. Struct. Biol..

[110] Atherton J., Moores C.A. (2021). Cryo-EM of kinesin-binding protein: Challenges and opportunities from protein-surface interactions. Acta Crystallogr. D. Struct. Biol..

[111] Oguievetskaia K., Martin-Chanas L., Vorotyntsev A., Doppelt-Azeroual O., Brotel X., Adcock S.A., de Brevern A.G., Delfaud F., Moriaud F. (2009). Computational fragment-based drug design to explore the hydrophobic sub-pocket of the mitotic kinesin Eg5 allosteric binding site. J. Comput. Aided Mol. Des..

[112] Zhang W., Zhai L., Lu W., Boohaker R.J., Padmalayam I., Li Y. (2016). Discovery of novel allosteric Eg5 inhibitors through structure-based virtual screening. Chem. Biol. Drug Des..

[113] Gao C., Lowndes N.F., Eriksson L.A. (2017). Analysis of biphenyl-type inhibitors targeting the Eg5 α4/α6 allosteric pocket. ACS Omega.

[114] Huang R., Oh H., Arrendale A., Martin V.A., Galan J., Workman E.J., Stout J.R., Walczak C.E., Tao W.A., Borch R.F. (2013). Intracellular targets for a phosphotyrosine peptidomimetic include the mitotic kinesin, MCAK. Biochem. Pharmacol..

[115] Yang Y., Zhang S., Wu Z., Li W., Sun X., Xuan Y., Hang T., Xu L., Chen X. (2025). Crystal structures of KIF2A complexed with WDR5 reveal the structural plasticity of WIN-S7 sites. Acta Biochim. Biophys. Sin..

[116] Bodun D.S., Ibrahim I.O., Bashiru M., Abolade R.O., Ndarawit W.K., John-Joy Owolade A., Temitope A., Somtochukwu I.J., Olugbogi E.A., Samadhi K.G.A. (2025). De novo design and bioactivity prediction of mitotic kinesin Eg5 inhibitors using MPNN and LSTM-based transfer learning. Comput. Biol. Med..

[117] Wang Y., Wu X., Du M., Chen X., Ning X., Chen H., Wang S., Liu J., Liu Z., Li R. (2017). Eg5 inhibitor YL001 induces mitotic arrest and inhibits tumor proliferation. Oncotarget.

[118] Qian Y., Wang Q., Yin L., Lu A.-Y. (2025). CF-DTI: Coarse-to-fine feature extraction for enhanced drug-target interaction prediction. Health Inf. Sci. Syst..

[119] Hijazo-Pechero S., Alay A., Cordero D., Marín R., Vilariño N., Palmero R., Brenes J., Montalban-Casafont A., Nadal E., Solé X. (2024). Transcriptional analysis of landmark molecular pathways in lung adenocarcinoma results in a clinically relevant classification with potential therapeutic implications. Mol. Oncol..

[120] Zhang X., Gao G., Zhang Q., Zhao S., Li X., Cao W., Luo H., Zhou C. (2024). In-depth proteomic analysis identifies key gene signatures predicting therapeutic efficacy of anti-PD-1/PD-L1 monotherapy in non-small cell lung cancer. Transl. Lung Cancer Res..

[121] Giliberto M., Santana L.M., Holien T., Misund K., Nakken S., Vodak D., Hovig E., Meza-Zepeda L.A., Coward E., Waage A. (2022). Mutational analysis and protein profiling predict drug sensitivity in multiple myeloma cell lines. Front. Oncol..

[122] Buttarelli M., Ciucci A., Palluzzi F., Raspaglio G., Marchetti C., Perrone E., Minucci A., Giacò L., Fagotti A., Scambia G. (2022). Identification of a novel gene signature predicting response to first-line chemotherapy in BRCA wild-type high-grade serous ovarian cancer patients. J. Exp. Clin. Cancer Res..

[123] Liu Q., Yuan W., Zhaowang R., Yuan X., Sun M. (2026). Copy-number amplification drives *IFI30* overexpression and coordinated immune activation, identifying a novel diagnostic and therapeutic target in gastric adenocarcinoma. Sci. Rep..

[124] Li X., Jiao Y., Liu Y. (2025). Precision medicine advances in pancreatic cancer driven by genomic and molecular alterations. World J. Gastrointest. Oncol..

[125] Qasim H., Abu Shugaer M., Awawdeh A.N., Dawaymeh T., Khattab K., Al-Oweiwi M., Leoni M.L.G., Varrassi G. (2026). Gastrointestinal stromal tumors: Histopathological spectrum, molecular subtypes, and implications for targeted therapy. Cureus.

[126] Kendre G., Marhenke S., Lorz G., Becker D., Reineke-Plaa T., Poth T., Murugesan K., Kühnel F., Woller N., Wirtz R.M. (2021). The co-mutational spectrum determines the therapeutic response in murine FGFR2 fusion-driven cholangiocarcinoma. Hepatology.

[127] Cao Z., Guan M., Cheng C., Wang F., Jing Y., Zhang K., Jiao J., Ruan L., Chen Z. (2024). *KIF20B* and *MET*, hub genes of *DIAPHs*, predict poor prognosis and promote pancreatic cancer progression. Pathol. Res. Pract..

[128] Xu M., Chen J., Xu Y., Zhang J.L., Zhou Y., He J.J., Wu S., Wei Y.L., She Z.Y. (2023). Generation of centromere-associated protein-E CENP-E-/- knockout cell lines using the CRISPR/Cas9 system. J. Vis. Exp..

[129] Zhang C., Wu B.Z., Di Ciano-Oliveira C., Wu Y.F., Khavkine Binstock S.S., Soria-Bretones I., Pham N., Elia A.J., Chari R., Lam W.L. (2024). Identification of *KIFC1* as a putative vulnerability in lung cancers with centrosome amplification. Cancer Gene Ther..

[130] Xiao M., Tang R., Pan H., Yang J., Tong X., Xu H., Guo Y., Lei Y., Wu D., Lei Y. (2025). TPX2 serves as a novel target for expanding the utility of PARPi in pancreatic cancer through conferring synthetic lethality. Gut.

[131] Qian X., DeGennaro E.M., Talukdar M., Akula S.K., Lai A., Shao D.D., Gonzalez D., Marciano J.H., Smith R.S., Hylton N.K. (2022). Loss of non-motor kinesin *KIF26A* causes congenital brain malformations via dysregulated neuronal migration and axonal growth as well as apoptosis. Dev. Cell.

[132] Li W., Cheng T., Dong X., Chen H., Yang L., Qiu Z., Zhou W. (2022). *KIF5C* deficiency causes abnormal cortical neuronal migration, dendritic branching, and spine morphology in mice. Pediatr. Res..

[133] Lu X., Que Y., Yang J., Le L., Cai Q., Xu B., Hong D., Liang Y., Zhang X. (2024). Targeting *KIFC1* promotes senescence in soft tissue sarcoma via FXR1-dependent regulation of MAD2L1 mRNA stability. Adv. Sci..

[134] Ye X.S., Fan L., Van Horn R.D., Nakai R., Ohta Y., Akinaga S., Murakata C., Yamashita Y., Yin T., Credille K.M. (2015). A novel Eg5 inhibitor (LY2523355) causes mitotic arrest and apoptosis in cancer cells and shows potent antitumor activity in xenograft tumor models. Mol. Cancer Ther..

[135] Murase Y., Ono H., Ogawa K., Yoshioka R., Ishikawa Y., Ueda H., Akahoshi K., Ban D., Kudo A., Tanaka S. (2021). Inhibitor library screening identifies ispinesib as a new potential chemotherapeutic agent for pancreatic cancers. Cancer Sci..

[136] Wei N., Yu Y., Yang Y., Wang X., Zhong Z., Chen X., Yu Y. (2022). Inhibition and down-regulation of motor protein Eg5 expression in primary sensory neurons reveal a novel therapeutic target for pathological pain. Neurotherapeutics.

[137] Nanes B.A., Bhatt K., Boujemaa-Paterski R., Azarova E., Munawar S., Rajendran D., Isogai T., Dean K.M., Medalia O., Danuser G. (2024). Keratin isoform shifts modulate motility signals during wound healing. bioRxiv.

[138] Liu M., Ran J., Zhou J. (2018). Non-canonical functions of the mitotic kinesin Eg5. Thorac. Cancer.

[139] Sherin L., Farwa S., Sohail A., Li Z., Bég O.A. (2018). Cancer drug therapy and stochastic modeling of “nano-motors”. Int. J. Nanomed..

[140] Tawfik H.O., El-Moselhy T.F., El-Din N.S., El-Hamamsy M.H. (2019). Design, synthesis, and bioactivity of dihydropyrimidine derivatives as kinesin spindle protein inhibitors. Bioorg. Med. Chem..

[141] Wacker S.A., Kashyap S., Li X., Kapoor T.M. (2011). Examining the mechanism of action of a kinesin inhibitor using stable isotope labeled inhibitors for cross-linking (SILIC). J. Am. Chem. Soc..

[142] Nikanjam M., Kurzrock R. (2025). Cracking the resistance code: The molecular reclassification of cancer and precision therapy strategies. Semin. Cancer Biol..

[143] Schneider J.L., Shaverdashvili K., Mino-Kenudson M., Digumarthy S.R., Do A., Liu A., Gainor J.F., Lennerz J.K., Burns T.F., Lin J.J. (2023). Lorlatinib and capmatinib in a ROS1-rearranged NSCLC with *MET*-driven resistance: Tumor response and evolution. npj Precis. Oncol..

[144] Gupta S., Patni K., Kaur S., Dhanjal J.K. (2025). Deciphering context-specific Axitinib escape pathways via multi-omics and explainable machine learning. J. Transl. Med..

[145] Sung H.W., Choi S., Chu C.H., Ouyang M., Kalyan S., Scott N., Hur S.C. (2022). Sensitizing drug-resistant cancer cells from blood using microfluidic electroporator. PLoS ONE.

[146] Wang S., Shu J., Wang N., He Z. (2025). Exosomal non-coding RNAs: Mediators of crosstalk between cancer and cancer stem cells. Cell Death Discov..

[147] Conteduca V., Scarpi E., Caroli P., Lolli C., Gurioli G., Brighi N., Poti G., Farolfi A., Altavilla A., Schepisi G. (2022). Combining liquid biopsy and functional imaging analysis in metastatic castration-resistant prostate cancer helps predict treatment outcome. Mol. Oncol..

[148] Liu J., Tian Y., Yi L., Gao Z., Lou M., Yuan K. (2022). High *KIF11* expression is associated with poor outcome of NSCLC. Tumori J..

[149] Liu Y., Zhan P., Zhou Z., Xing Z., Zhu S., Ma C., Li Q., Zhu Q., Miao Y., Zhang J. (2016). The overexpression of *KIFC1* was associated with the proliferation and prognosis of non-small cell lung cancer. J. Thorac. Dis..

[150] Daigo K., Takano A., Thang P.M., Yoshitake Y., Shinohara M., Tohnai I., Murakami Y., Maegawa J., Daigo Y. (2018). Characterization of *KIF11* as a novel prognostic biomarker and therapeutic target for oral cancer. Int. J. Oncol..

[151] Jin Q., Dai Y., Wang Y., Zhang S., Liu G. (2019). High kinesin family member 11 expression predicts poor prognosis in patients with clear cell renal cell carcinoma. J. Clin. Pathol..

[152] Zhou Y., Chen X., Li B., Li Y., Zhang B. (2023). *KIF11* is a potential prognostic biomarker and therapeutic target for adrenocortical carcinoma. Transl. Androl. Urol..

[153] Wang B., Yu J., Sun Z., Luh F., Lin D., Shen Y., Wang T., Zhang Q., Liu X. (2020). Kinesin family member 11 is a potential therapeutic target and is suppressed by microRNA-30a in breast cancer. Mol. Carcinog..

[154] Piao X., Byun Y.J., Jeong P., Ha Y., Yoo E.S., Yun S.J., Kim W. (2017). Kinesin family member 11 mRNA expression predicts prostate cancer aggressiveness. Clin. Genitourin. Cancer.

[155] Mo X., Zhang Z., Song M., Zhou Z., Zeng J., Du Y., Sun F., Yang J., He J., Huang Y. (2021). Screening and identification of hub genes in bladder cancer by bioinformatics analysis and *KIF11* is a potential prognostic biomarker. Oncol. Lett..

[156] Thériault B.L., Pajovic S., Bernardini M.Q., Shaw P.A., Gallie B.L. (2012). Kinesin family member 14: An independent prognostic marker and potential therapeutic target for ovarian cancer. Int. J. Cancer.

[157] Wang W., Shi Y., Li J., Cui W., Yang B. (2016). Up-regulation of *KIF14* is a predictor of poor survival and a novel prognostic biomarker of chemoresistance to paclitaxel treatment in cervical cancer. Biosci. Rep..

[158] Corson T.W., Gallie B.L. (2006). *KIF14* mRNA expression is a predictor of grade and outcome in breast cancer. Int. J. Cancer.

[159] Wang Q., Wang L., Li D., Deng J., Zhao Z., He S., Zhang Y., Tu Y. (2013). Kinesin family member 14 is a candidate prognostic marker for outcome of glioma patients. Cancer Epidemiol..

[160] Zhang Y., Yuan Y., Liang P., Zhang Z., Guo X., Xia L., Zhao Y., Shu X., Sun S., Ying Y. (2017). Overexpression of a novel candidate oncogene *KIF14* correlates with tumor progression and poor prognosis in prostate cancer. Oncotarget.

[161] Nakamura M., Takano A., Thang P.M., Tsevegjav B., Zhu M., Yokose T., Yamashita T., Miyagi Y., Daigo Y. (2020). Characterization of *KIF20A* as a prognostic biomarker and therapeutic target for different subtypes of breast cancer. Int. J. Oncol..

[162] Kawai Y., Shibata K., Sakata J., Suzuki S., Utsumi F., Niimi K., Sekiya R., Senga T., Kikkawa F., Kajiyama H. (2018). *KIF20A* expression as a prognostic indicator and its possible involvement in the proliferation of ovarian clear-cell carcinoma cells. Oncol. Rep..

[163] Li H., Zhang W., Sun X., Chen J., Li Y., Niu C., Xu B., Zhang Y. (2018). Overexpression of kinesin family member 20A is associated with unfavorable clinical outcome and tumor progression in epithelial ovarian cancer. Cancer Manag. Res..

[164] Liu S., Lin H., Qiu F., Zhang W., Niu C., Wen W., Sun X., Ye L., Wu X., Lin C. (2017). Overexpression of kinesin family member 20A correlates with disease progression and poor prognosis in human nasopharyngeal cancer: A retrospective analysis of 105 patients. PLoS ONE.

[165] Shen T., Yang L., Zhang Z., Yu J., Dai L., Gao M., Shang Z., Niu Y. (2019). *KIF20A* affects the prognosis of bladder cancer by promoting the proliferation and metastasis of bladder cancer cells. Dis. Markers.

[166] Fan X., Wang X., Zhu H., Wang W., Zhang S., Wang Z. (2015). *KIF2A* overexpression and its association with clinicopathologic characteristics and unfavorable prognosis in colorectal cancer. Tumour Biol..

[167] Wang D., Zhu H., Ye Q., Wang C., Xu Y. (2016). Prognostic value of *KIF2A* and HER2-Neu overexpression in patients with epithelial ovarian cancer. Medicine.

[168] Lei G., Xin X., Hu X. (2022). Clinical significance of kinesin family member 2A as a facilitating biomarker of disease surveillance and prognostication in cervical cancer patients. Ir. J. Med. Sci..

[169] Zhang S., Huang F., Wang Y., Song Q., Yang X., Wu H. (2016). *KIF2A* overexpression and its association with clinicopathologic characteristics and poor prognoses in patients with gastric cancer. Dis. Markers.

[170] Zhang X., Wu M., Peng G., Li W., Guo Z., Li H., Jiang M. (2022). Aberrant kinesin family member 2A signifies tumor size and invasion, and may help predict prognosis of patients with papillary thyroid carcinoma. Oncol. Lett..

[171] Mo S., Fang D., Zhao S., Thai Hoa P.T., Zhou C., Liang T., He Y., Yu T., Chen Y., Qin W. (2022). Downregulated oncogene *KIF2C* inhibits growth, invasion, and metastasis of hepatocellular carcinoma through the Ras/MAPK signaling pathway and epithelial-to-mesenchymal transition. Ann. Transl. Med..

[172] Kreis N.N., Moon H.H., Wordeman L., Louwen F., Solbach C., Yuan J., Ritter A. (2024). *KIF2C*/MCAK a prognostic biomarker and its oncogenic potential in malignant progression, and prognosis of cancer patients: A systematic review and meta-analysis as biomarker. Crit. Rev. Clin. Lab. Sci..

[173] Zhang P., Gao H., Ye C., Yan R., Yu L., Xia C., Yang D. (2022). Large-scale transcriptome data analysis identifies *KIF2C* as a potential therapeutic target associated with immune infiltration in prostate cancer. Front. Immunol..

[174] Li S., Ma Y., Wu C., Hou X. (2023). Knockdown of kinesin family member 2C restricts cell proliferation and induces cell cycle arrest in gastric cancer. Histol. Histopathol..

[175] Liu S., Ye Z., Xue V.W., Sun Q., Li H., Lu D. (2023). *KIF2C* is a prognostic biomarker associated with immune cell infiltration in breast cancer. BMC Cancer.

[176] Bie L., Zhao G., Wang Y., Zhang B. (2012). Kinesin family member 2C (*KIF2C*/MCAK) is a novel marker for prognosis in human gliomas. Clin. Neurol. Neurosurg..

[177] An L., Zhang J., Feng D., Zhao Y., Ouyang W., Shi R., Zhou X., Yu Z., Wei S., Min J. (2021). *KIF2C* is a novel prognostic biomarker and correlated with immune infiltration in endometrial cancer. Stem Cells Int..

[178] Wang C., Su Y., Shi J., Feng G. (2025). *KIF2C* promotes oral squamous cell carcinoma progression via PLK1 upregulation: Implications for biomarker development and therapeutic targeting. J. Mol. Histol..

[179] Taniwaki M., Takano A., Ishikawa N., Yasui W., Inai K., Nishimura H., Tsuchiya E., Kohno N., Nakamura Y., Daigo Y. (2007). Activation of *KIF4A* as a prognostic biomarker and therapeutic target for lung cancer. Clin. Cancer Res..

[180] Huang Y., Wang H., Lian Y., Wu X., Zhou L., Wang J., Deng M., Huang Y. (2018). Upregulation of kinesin family member 4A enhanced cell proliferation via activation of Akt signaling and predicted a poor prognosis in hepatocellular carcinoma. Cell Death Dis..

[181] Liu G., Lu Y., Li L., Jiang T., Chu S., Hou P., Bai J., Chen M. (2020). The kinesin motor protein *KIF4A* as a potential therapeutic target in renal cell carcinoma. Investig. New Drugs.

[182] Liu Y., Li Y., Tang C., Wen H., Tang J., Chen G., Wu Y. (2025). *KIF4A* in disease pathogenesis and therapeutics: From molecular mechanisms to clinical translation. Biol. Direct.

[183] Song X., Zhang T., Wang X., Liao X., Han C., Yang C., Su K., Cao W., Gong Y., Chen Z. (2018). Distinct diagnostic and prognostic values of kinesin family member gene expression in patients with breast cancer. Med. Sci. Monit..

[184] Kasahara M., Nagahara M., Nakagawa T., Ishikawa T., Sato T., Uetake H., Sugihara K. (2016). Clinicopathological relevance of kinesin family member 18A expression in invasive breast cancer. Oncol. Lett..

[185] Nan K., Zhang L., Zou Y., Geng Z., Huang J., Peng Y., Yin S., Zhang M. (2025). Integrated profiling delineated *KIF18A* as a significant biomarker associated with both prognostic outcomes and immune response in pancreatic cancer. Immunotargets Ther..

[186] Tao B., Liu Y., Liu H., Zhang Z., Guan Y., Wang H., Shi Y., Zhang J. (2022). Prognostic biomarker *KIF18A* and its correlations with immune infiltrates and mitosis in glioma. Front. Genet..

[187] Li X., Liu M., Zhang Z., Zhang L., Liang X., Sun L., Zhong D. (2019). High kinesin family member 18A expression correlates with poor prognosis in primary lung adenocarcinoma. Thorac. Cancer.

[188] Li D., Yu T., Han J., Xu X., Wu J., Song W., Liu G., Zhu H., Zeng Z. (2022). Prognostic value and immunological role of *KIFC1* in hepatocellular carcinoma. Front. Mol. Biosci..

[189] Li G., Chong T., Yang J., Li H., Chen H. (2018). Kinesin motor protein *KIFC1* is a target protein of miR-338-3p and is associated with poor prognosis and progression of renal cell carcinoma. Oncol. Res..

[190] Ding L., Li B., Yu X., Li Z., Li X., Dang S., Lv Q., Wei J., Sun H., Chen H. (2020). *KIF15* facilitates gastric cancer by enhancing proliferation, inhibiting apoptosis, and predicting poor prognosis. Cancer Cell Int..

[191] Sheng J., Li C., Dong M., Jiang K. (2020). Identification by comprehensive bioinformatics analysis of *KIF15* as a candidate risk gene for triple-negative breast cancer. Cancer Manag. Res..

[192] Qureshi Z., Ahmad M., Yang W., Tan F. (2021). Kinesin 12 (*KIF15*) contributes to the development and tumorigenicity of prostate cancer. Biochem. Biophys. Res. Commun..

[193] Sun Z., Pan F., Shao J., Yan Q., Lu L., Zhang N. (2020). Kinesin superfamily protein 21B acts as an oncogene in non-small cell lung cancer. Cancer Cell Int..

[194] Gong Y., Zhou L., Ding L., Zhao J., Wang Z., Ren G., Zhang J., Mao Z., Zhou R. (2022). *KIF23* is a potential biomarker of diffuse large B-cell lymphoma: Analysis based on bioinformatics and immunohistochemistry. Medicine.

[195] Sun L., Zhang C., Yang Z., Wu Y., Wang H., Bao Z., Jiang T. (2016). *KIF23* is an independent prognostic biomarker in glioma, transcriptionally regulated by TCF-4. Oncotarget.

