# Supplementary material for: Kinesins in Cancer Drug Resistance: Mechanisms, Therapeutic Targeting, and Translational Potential
Source: Cancers (Basel). 2026 Sep 5;18(17):2872. doi: 10.3390/cancers18172872 (PMC13564715; doi:10.3390/cancers18172872)
Supplement: Supplementary file 1 [file cancers-18-02872-s001.zip › cancers-4505429-supplementary.pdf]

Supplementary Table S1. Expression patterns and clinical associations of kinesin family members across human cancers

Kinesin	Cancer type	Expression pattern	Clinicopathological / prognostic association	Ref. no.
KIF11	NSCLC	Upregulated	Associated with lymph node metastasis, pathological stage, and poor OS	[31,148,149]
KIF11	Oral cancer	Upregulated	Independently associated with poor prognosis	[150]
KIF11	Clear cell renal cell carcinoma	Upregulated	Associated with nuclear grade, TNM stage, and poor prognosis	[151]
KIF11	Adrenocortical carcinoma	Upregulated	Associated with T/M stage, recurrence risk, and shorter OS	[152]
KIF11	Breast cancer	Upregulated	Associated with poor survival	[153]
KIF11	Prostate cancer	Upregulated	Associated with PSA, Gleason score, advanced stage, and metastasis	[154]
KIF11	Bladder cancer	Upregulated	Potential prognostic biomarker	[155]
KIF14	Ovarian cancer	Upregulated / genomic gain	Independently predicts worse outcome and recurrence	[156]
KIF14	Cervical cancer	Upregulated	Associated with stage, lymph-node metastasis, and poor OS	[157]
KIF14	Breast cancer	Upregulated	Associated with nodal involvement and poor OS/DFS	[158]
KIF14	Glioma	Upregulated	Increases with grade and predicts poor OS	[159]
KIF14	Prostate cancer	Upregulated	Associated with T stage, Gleason score, and shorter OS	[160]
KIF20A	Hepatocellular carcinoma	Upregulated	Associated with malignant progression and immune-related features	[75]
KIF20A	Breast cancer	Upregulated	Independent poor prognostic factor across subtypes	[161]
KIF20A	Ovarian clear cell carcinoma	Upregulated	Associated with poorer PFS/OS	[162]
KIF20A	Epithelial ovarian cancer	Upregulated	Associated with metastasis, recurrence, and poor OS/PFS	[163]
KIF20A	Nasopharyngeal carcinoma	Upregulated	Associated with stage, distant metastasis, and shorter OS/PFS	[164]
KIF20A	Glioma	Upregulated	Associated with glioma prognosis and malignant/EMT-related behavior	[41]
KIF20A	Bladder cancer	Upregulated	Associated with high grade, advanced stage, and poor prognosis	[165]
KIF2A	Colorectal cancer	Upregulated	Associated with TNM/T stage and poor prognosis	[166]
KIF2A	Ovarian epithelial cancer	Upregulated	Associated with lymph-node metastasis, FIGO stage, and worse OS	[167]

Kinesin	Cancer type	Expression pattern	Clinicopathological / prognostic association	Ref. no.
KIF2A	Cervical cancer	Upregulated	Associated with lymph-node metastasis, FIGO stage, and worse DFS/OS	[168]
KIF2A	Gastric cancer	Upregulated	Associated with TNM stage, lymph-node metastasis, and poor survival	[169]
KIF2A	Papillary thyroid carcinoma	Upregulated	Associated with tumor size, pTNM stage, and shorter DFS	[170]
KIF2C	Hepatocellular carcinoma	Upregulated	Associated with poor prognosis, grade, and stage	[171,172]
KIF2C	Prostate cancer	Upregulated	Associated with stage, lymph-node metastasis, PSA, Gleason score, and poor prognosis	[173]
KIF2C	Gastric cancer	Upregulated	Associated with diagnosis/prognosis and immune infiltration	[174]
KIF2C	Breast cancer	Upregulated	Associated with poor OS and immune infiltration	[175]
KIF2C	Glioma	Upregulated	Associated with histopathological grade and poor prognosis	[176]
KIF2C	Endometrial cancer	Upregulated	Prognostic and immune-related biomarker	[177]
KIF2C	Oral squamous cell carcinoma	Upregulated	Potential prognostic biomarker and therapeutic-target candidate	[178]
KIF4A	NSCLC	Upregulated	Independent poor prognostic marker; associated with shorter survival	[179]
KIF4A	Hepatocellular carcinoma	Upregulated	Associated with poorer OS and DFS	[180]
KIF4A	Renal cell carcinoma	Upregulated	Associated with aggressive features and poor OS/DFS	[181]
KIF4A	Breast / lung / liver / colorectal / ovarian cancers	Upregulated	Broadly associated with poor clinical outcome	[182]
KIF18A	Breast cancer	Upregulated	Associated with lymph-node metastasis and poor DFS	[183]
KIF18A	ER-positive breast cancer	Upregulated	Associated with poor RFS, DMFS, and breast cancer-specific survival	[184]
KIF18A	Pancreatic adenocarcinoma	Upregulated	Associated with worse stage and poor prognosis	[185]
KIF18A	Glioma	Upregulated	Independent prognostic factor	[186]
KIF18A	Lung adenocarcinoma	Upregulated	Associated with poor differentiation, nodal metastasis, and shorter DFS/OS	[187]
KIFC1	Pan-cancer	Upregulated	Broadly associated with adverse outcomes	[13]
KIFC1	Prostate cancer	Upregulated	Associated with higher Gleason score, stage, metastasis, and shorter RFS	[24]
KIFC1	Hepatocellular carcinoma	Upregulated	Correlated with emboli, metastasis, recurrence; independent prognostic marker	[188]
KIFC1	Renal cell carcinoma	Upregulated	Associated with aggressive clinicopathological features and poor survival	[189]

Kinesin	Cancer type	Expression pattern	Clinicopathological / prognostic association	Ref. no.
KIFC1	Glioma	Upregulated	Associated with tumor grade and poor prognosis	[28]
KIFC1	NSCLC / lung cancer	Upregulated	Associated with poor OS	[27,129,149]
KIF15	Gastric cancer	Upregulated	High expression predicts poor prognosis	[190]
KIF15	Triple-negative breast cancer	Upregulated	Associated with aggressive behavior; biomarker/target candidate	[191]
KIF15	Prostate cancer	Upregulated	Associated with tumor growth and proposed as a potential therapeutic target	[192]
KIF20B	Breast cancer	Upregulated	Associated with advanced nodal stage and independently predicts poor prognosis	[22]
KIF21B	NSCLC	Upregulated	Associated with nodal status, pTNM stage, and poor prognosis	[193]
KIF23	DLBCL	Upregulated	Independent adverse prognostic factor	[194]
KIF23	Glioma	Upregulated	Associated with high grade and worse survival	[195]
KIF26B	Colorectal cancer	Upregulated	Associated with stage and poor OS	[21]
KIF17	Breast cancer	Downregulated	Reduced expression is associated with worse outcome; tumor-suppressive pattern	[29]
KIF3A	NSCLC	Downregulated	Reduced expression is associated with poor survival; tumor-suppressive pattern	[30]
